# MAIT cell-MR1 reactivity is highly conserved across multiple divergent species

**DOI:** 10.1016/j.jbc.2024.107338

**Published:** 2024-05-03

**Authors:** Matthew D. Edmans, Timothy K. Connelley, Sophie Morgan, Troi J. Pediongco, Siddharth Jayaraman, Jennifer A. Juno, Bronwyn S. Meehan, Phoebe M. Dewar, Emmanuel A. Maze, Eduard O. Roos, Basudev Paudyal, Jeffrey Y.W. Mak, Ligong Liu, David P. Fairlie, Huimeng Wang, Alexandra J. Corbett, James McCluskey, Lindert Benedictus, Elma Tchilian, Paul Klenerman, Sidonia B.G. Eckle

**Affiliations:** 1 Department of Enhanced Host Responses, The Pirbright Institute, Pirbright, United Kingdom; 2 Peter Medawar Building for Pathogen Research, University of Oxford, Oxford, United Kingdom; 3 Division of Infection and Immunity, The Roslin Institute, The University of Edinburgh, Roslin, United Kingdom; 4 Department of Microbiology and Immunology, Peter Doherty Institute for Infection and Immunity, The University of Melbourne, Melbourne, Victoria, Australia; 5 Centre for Chemistry and Drug Discovery, Institute for Molecular Bioscience, The University of Queensland, Brisbane, Queensland, Australia; 6 Australian Research Council Centre of Excellence for Innovations in Peptide and Protein Science, Institute for Molecular Bioscience, The University of Queensland, Brisbane, Queensland, Australia; 7 State Key Laboratory of Respiratory Disease, Guangzhou Institute of Respiratory Disease, Guangzhou Medical University, Guangzhou, China; 8 Faculty of Veterinary Medicine, Department of Population Health Sciences, Utrecht University, Utrecht, The Netherlands

**Keywords:** antigen (Ag), 5-(2-oxopropylideneamino)-6-d-ribitylaminouracil (5-OP-RU), major histocompatibility complex (MHC), MHC-I related protein 1 (MR1), T cell receptor (TCR), mucosal-associated invariant T (MAIT) cell, comparative immunology, innate-like immunity, T cell biology

## Abstract

Mucosal-associated invariant T (MAIT) cells are a subset of unconventional T cells that recognize small molecule metabolites presented by major histocompatibility complex class I related protein 1 (MR1), *via* an αβ T cell receptor (TCR). MAIT TCRs feature an essentially invariant TCR α-chain, which is highly conserved between mammals. Similarly, MR1 is the most highly conserved major histocompatibility complex-I–like molecule. This extreme conservation, including the mode of interaction between the MAIT TCR and MR1, has been shown to allow for species-mismatched reactivities unique in T cell biology, thereby allowing the use of selected species-mismatched MR1–antigen (MR1–Ag) tetramers in comparative immunology studies. However, the pattern of cross-reactivity of species-mismatched MR1–Ag tetramers in identifying MAIT cells in diverse species has not been formally assessed. We developed novel cattle and pig MR1–Ag tetramers and utilized these alongside previously developed human, mouse, and pig-tailed macaque MR1–Ag tetramers to characterize cross-species tetramer reactivities. MR1–Ag tetramers from each species identified T cell populations in distantly related species with specificity that was comparable to species-matched MR1–Ag tetramers. However, there were subtle differences in staining characteristics with practical implications for the accurate identification of MAIT cells. Pig MR1 is sufficiently conserved across species that pig MR1–Ag tetramers identified MAIT cells from the other species. However, MAIT cells in pigs were at the limits of phenotypic detection. In the absence of sheep MR1–Ag tetramers, a MAIT cell population in sheep blood was identified phenotypically, utilizing species-mismatched MR1–Ag tetramers. Collectively, our results validate the use and define the limitations of species-mismatched MR1–Ag tetramers in comparative immunology studies.

Mucosal-associated invariant T (MAIT) cells are a subset of unconventional, innate-like T cells (1, 2) that are restricted by the major histocompatibility complex class I (MHC-I)-like antigen presenting molecule MHC-I related protein 1 (MR1) (3, 4) associated with β2 microglobulin (β2m) (5, 6, 7). MAIT cells recognize small molecule metabolites presented by MR1 *via* their αβ T cell receptor (TCR) (7, 8). The most potent antigens are 5-(2-oxopropylideneamino)-6-d-ribitylaminouracil (5-OP-RU) and 5-(2-oxoethylideneamino)-6-d-ribitylaminouracil, derived from the condensation of methylglyoxal and glyoxal, respectively, with the riboflavin biosynthesis intermediate 5-amino-6-d-ribitylaminouracil produced by many bacteria and fungi (8). Recognition of these antigens, either produced by microbes or chemically synthesized, triggers MAIT cells to mount a potent and polyfunctional immune response (8, 9, 10), reviewed in (11, 12). Indeed, MAIT cells contribute to protective immunity against some, but not all riboflavin-producing microbes, such as *Francisella tularensis* (13, 14), *Legionella longbeachae* (15), *Klebsiella pneumoniae* (16), *Escherichia coli* (9), and *Mycobacterium tuberculosis* and *M. bovis* BCG (9, 17, 18, 19, 20, 21). MAIT cells have also been shown to play a role in tissue repair (22, 23, 24, 25, 26, 27) and homeostasis (28).

The *MR1* gene is found in most placental mammals, including humans (located on Chr. 1) (4), mice (Chr. 1) (4, 29), rats (Chr. 13) (30), cattle (Chr. 16) (31, 32, 33), sheep (Chr. 12) (31, 33), pigs (Chr. 9) (33, 34), and bats (Chr. unknown) (35) as well as non-human primates (36, 37) and marsupials (38) but not in other lower jawed vertebrates, including cartilaginous fish (*e.g.*, shark), teleosts (*e.g.*, zebrafish), and monotremes (*e.g.*, platypus) (38). The conserved synteny within the gene organization and chromosomal location outside the *MHC* locus (29, 31, 33, 34, 38, 39) suggests that *MR1* was established in a common ancestor of mammalian species (4) and, more precisely, of placental and marsupial mammals after divergence from the monotremes about 170 million years ago (38, 40). The *MR1* gene exists as a single copy and, depending on the species, is mono-/oligomorphic (29, 33, 37, 38, 39, 41, 42, 43, 44, 45, 46). Furthermore, between species, and particularly in the ligand-binding α1-/α2-domains, where synonymous substitutions predominate (32), the amino acid sequence of MR1 is more identical than any other MHC-I–like, classical, or non-classical MHC molecules that have an antigen-presenting function (4, 30, 33, 38). For instance, human and mouse MR1 are 83.8% and 89.4% identical, considering the α1-/α2-/α3-domains or only the α1-/α2-domains, respectively. This is suggestive of a strong, purifying (*i.e.*, against diversification) evolutionarily selective pressure maintaining *MR1* (29, 40).

A key hallmark of MAIT cells is the expression of an essentially invariant TCR α-chain involving the rearrangement between the *TCR α*-chain variable *(TRAV)* and joining *(TRAJ)* gene segments *TRAV1-2* and *TRAJ33* (1) or at lower frequency *TRAJ12* or *TRAJ20* in humans (47, 48, 49). Through these rearrangements, a CDR3α-loop of constant length is formed featuring a nearly invariant sequence with a tyrosine residue at position 95 (1, 2, 47, 48, 49). This is the MAIT TCR residue most crucial to ligand binding: it is the only TCR residue that directly contacts the antigen (2′-OH group of 5-OP-RU) while simultaneously contacting the MR1 residue Y152, *via* hydrogen bonds (8, 50, 51). Indeed, the MAIT TCR Y95α residue, the MR1 Y152 residue, and 5-OP-RU form an ‘interaction triad’ (52). The dominant human MAIT TCR α-chain, *TRAV1-2-TRAJ33*, is evolutionarily highly conserved among mammals, where it is rearranged from homologous *TRAV* and *TRAJ* elements (*TRAV1-TRAJ33*) forming a CDR3α-loop of constant length, as identified in mice (2), cattle (2, 19), sheep (31), macaques (53, 54), and pigs (55). Based on an analysis of available genomic data, homologs of the *TRAV1* and *TRAJ33* genes are present in most mammals, including marsupials (38). Interestingly, the *TRAV1* gene segment is missing from the genome of some placental mammals, including carnivores, lagomorphs as well as in the armadillo, a xenarthran (40). In these latter species, the *MR1* gene is also lost or inactivated, suggesting that the *MR1* and *TRAV1* genes coevolved in mammals (40).

There is also some restriction in the *TRBV* gene segment usage of MAIT TCRs (1, 2, 55) and, between species, this can be biased to orthologous *TRBV* gene segments. For instance, in humans, there is a bias to *TRBV20* and *TRBV6* (1, 2); in cattle to *TRBV4*, *TRBV7*, and *TRBV20*, with *TRBV20* being the cattle orthologous segment of human *TRBV20* (19), and in mice to *TRBV19* and *TRBV13*, both being the murine orthologous segments of human *TRBV6* (2). However, there is no obvious restriction in *TRBJ* usage, no preferred amino acid motif (2, 48), and limited bias in the CDR3β-loop length (48). Thus, MAIT TCRs have been described as “semi-invariant” (2).

Fluorescently labeled MR1 tetramers loaded with the MAIT cell antigen 5-OP-RU represent the gold standard for identifying MAIT cells in blood and tissues (8, 49). The folic acid photodegradation product 6-formylpterin (6-FP) (7) and its acetylated analog, Acetyl-6-FP (Ac-6-FP) (51), also bind to MR1 but do not stimulate MAIT cells (7, 51, 56). Similarly, MR1 tetramers loaded with 6-FP or Ac-6-FP typically do not bind to MAIT cells and thus are used as negative controls of MR1–5-OP-RU tetramer staining (in humans (57)) or as a blocking reagent during MR1–5-OP-RU tetramer staining (in mice (58)). Of note, based on MR1–antigen (MR1–Ag) tetramer staining, some human MAIT cells and non-MAIT, MR1-reactive T cells have been identified in humans and mice that can variably cross-react with 6-FP-, Ac-6-FP-, and 3-formylsalicylic acid–loaded MR1 tetramers (59, 60), reviewed in (61). However, the binding of these MR1–Ag tetramers to T cells is generally dependent upon CD8 (59, 60), suggesting the intrinsic affinity of the TCR-MR1–Ag complex may be functionally suboptimal.

MAIT cell biology has primarily been studied in humans (8, 49, 62), mice (15, 63), and macaques (54, 64) and more recently in cattle and opossum (65) using species-matched MR1–Ag tetramer reagents. Broad comparative immunology studies of MAIT cells will help inform their evolutionary conserved role and function. Greater fundamental understanding of MAIT cell biology in a range of species will assist with vaccine and therapeutic approaches relevant to human and animal health (66). However, such studies are currently hindered by the limited availability and understanding of species matched MR1–Ag tetramers for MAIT cell identification beyond those from human, mouse, and macaque. Due to the phylogenetic conservation of the MAIT–MR1 axis, recognition of MAIT cells from disparate species by orthologous MR1–Ag tetramers represents an alternative approach which has been used for the identification and characterization of MAIT cells in multiple species of macaques (53, 54, 67), bats (35), cattle (19), rats (65), and sheep (65). However, the specificity and sensitivity with which species-mismatched MR1–Ag tetramers identify MAIT cells and the use of this approach for other species has not been formally assessed.

Here, we utilized newly developed cattle and pig MR1–5-OP-RU and MR1–6-FP tetramers, in addition to existing human, mouse, and pig-tailed macaque MR1–5-OP-RU and MR1–6-FP tetramers, to identify MAIT cells in different species and to examine evolutionary conservation based on cross-species MR1-tetramer reactivities, a unique opportunity provided by the MAIT–MR1 axis. MR1–Ag tetramers broadly cross-reacted with MAIT cells from distantly related species consistent with the high conservation of the MAIT–MR1 axis across species. Moreover, this approach identified MAIT cells phenotypically in sheep and demonstrated that MAIT cells were at the lower limit of phenotypic detection in pigs. However, a systematic comparison of species-matched and species-mismatched MR1–Ag tetramer staining across species exposed different levels of cross-reactivities in each species that manifested in differences in the frequencies of MR1–Ag tetramer^+^ T cells identified, the mean and range of MR1–Ag tetramer-staining intensity of MAIT cells (a surrogate for avidity), and variation in the patterns of antigen specificity. Our results point to subtle differences in the MAIT–MR1 axis between species with practical implications in the use of species-mismatched MR1–Ag tetramer staining in comparative immunology studies.

## Results

### Generation and characterization of pig- and cattle-specific MR1–5-OP-RU and MR1–6-FP monomers

**Figure 1:**
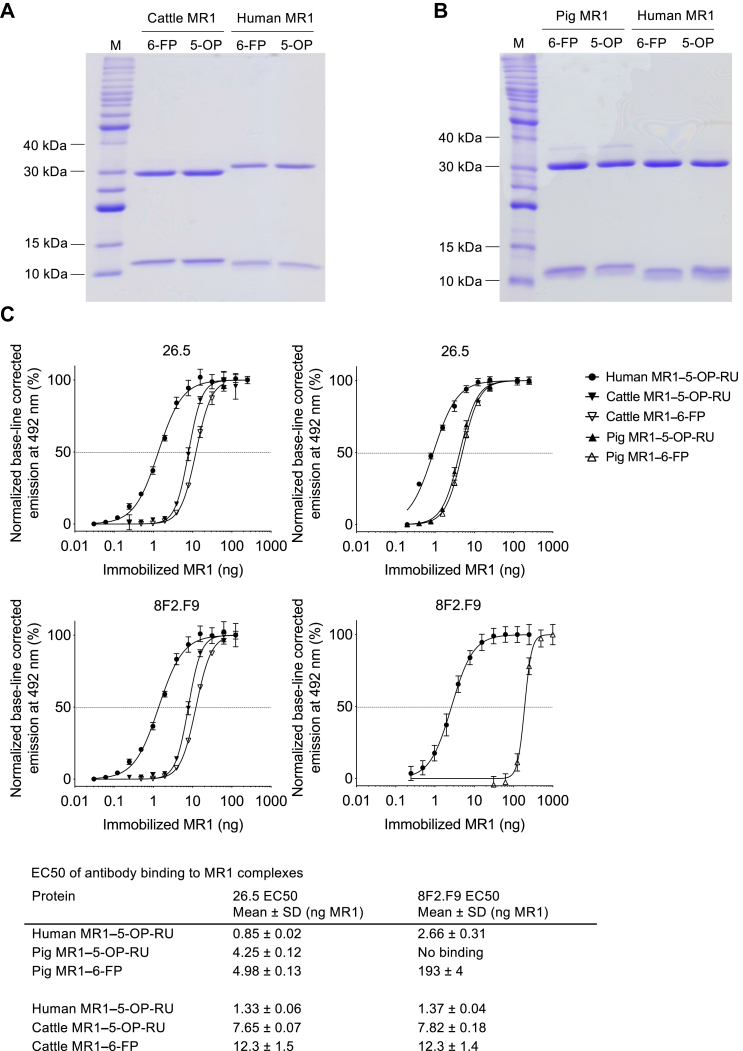
**Biochemical characterization of recombinant cattle and pig MR1–5-OP-RU and MR1–6-FP monomers.***A* and *B*, 15% SDS-PAGE under non-reducing conditions of 1.5 μg purified biotinylated cattle, pig, and human MR1 in complex with β2m and loaded with 5-OP-RU (5-OP) or 6-FP in comparison to a protein ladder (M) with molecular weights of proteins indicated as relevant. Accounting for loss of 4 H atoms and 2 H atoms due to the formation of two disulphide bonds in MR1 and one disulphide bond in β2m; the molecular weights of biotinylated MR1 and β2m are as follows: human: MR1: 32,258 Da, β2m: 11,860 Da; cattle MR1: 32403 Da, β2m: 11764 Da; pig MR1: 32526 Da, β2m: 11542 Da. *C*, 5-OP-RU- and 6-FP-loaded cattle and pig MR1 monomers in comparison to biotinylated human MR1–5-OP-RU (assessed previously alongside other species’ MR1 molecules, (64)) in ELISA with mAbs 26.5 and 8F2.F9 showing normalized, base-line corrected dose-response curves (n = 3, mean ± SD). EC_50_ values, as summarized in the table, were determined based on non-linear curve fits shown in the charts. 5-OP-RU, 5-(2-oxopropylideneamino)-6-d-ribitylaminouracil; 6-FP, 6-formylpterin; β2m, β2 microglobulin; MR1, MHC-I related protein 1.

Biotinylated *Sus scrofa* (pig) and *Bos taurus* (cattle) MR1 monomers loaded with synthetic 5-OP-RU or 6-FP were generated, as described previously for human, mouse, and pig-tailed macaque MR1–Ag monomers (8, 49, 64). Biotinylated pig and cattle MR1 monomers had the predicted molecular weights by gel electrophoresis and were of high purity, comparable to human biotinylated MR1 monomers (Fig. 1, *A* and *B*). The conformation-dependent mAb clone 26.5, specific for human MR1 (68), cross-reacts in flow cytometric staining with mouse, rat, cattle (32, 68), and pig (34) MR1, as well as reacting in ELISA with human, mouse, and pig-tailed macaque recombinant MR1 (7, 64). These findings are consistent with the high conservation of MR1 across mammalian species (29, 33, 38). The mouse MR1-specific (69) mAb clone 8F2.F9 is also conformation-dependent (70) but binds a spatially distinct epitope compared to the mAb 26.5 (69) encompassing residues of the α1-helix (K78) and neighboring loops (I16, H17) of the β-sheets located outside the binding groove proximal to the F′-pocket (69) (Fig. S1, *A* and *C*). Both mAb 26.5 and mAb 8F2.F9 cross-react in flow cytometry with rat, cattle, and human MR1 (69) and in ELISA with human, mouse, and pig-tailed macaque MR1 (64). Thus, we used the mAbs 26.5 and 8F2.F9 to assess the conformational integrity of the biotinylated pig and cattle MR1–Ag monomers, which differ from the human MR1 α1- and α2-domains in 20 and 28 amino acids, respectively (Fig. S1, *A*, *D*, *F*, and *G*). Indeed, both mAbs 26.5 and 8F2.F9 specifically bound cattle MR1–5-OP-RU and MR1–6-FP in a dose-dependent manner with EC_50_ ELISA values, ∼6- and ∼9-fold higher, respectively, than those observed for human MR1–5-OP-RU monomer and as reported previously (64), (Figs. 1*C*, and S2, *A* and *B*). The same applied to mAb 26.5 binding to pig MR1–5-OP-RU and MR1–6-FP (EC_50_ ELISA values ∼5- and ∼6-fold higher, respectively, than those observed for human MR1–5-OP-RU) (Figs. 1*C*, and S2*C*). However, the mAb 8F2.F9 bound poorly to pig MR1–6-FP (EC_50_ ELISA value ∼73-fold higher than that observed for human MR1–5-OP-RU) and binding was not observed to pig MR1–5-OP-RU (Figs. 1C, and S2*D*). Given that biotinylated pig and cattle MR1–5-OP-RU and MR1–6-FP monomers were recognized by at least one MR1-specific mAb, we considered the conformational integrity of these proteins was intact and proceeded to generate fluorochrome-tagged MR1–5-OP-RU and MR1–6-FP tetramers for flow cytometric analysis of MAIT cells in the different species.

### Identification of MAIT cells in multiple species using species-matched MR1–5-OP-RU and MR1–6-FP tetramers

**Figure 2:**
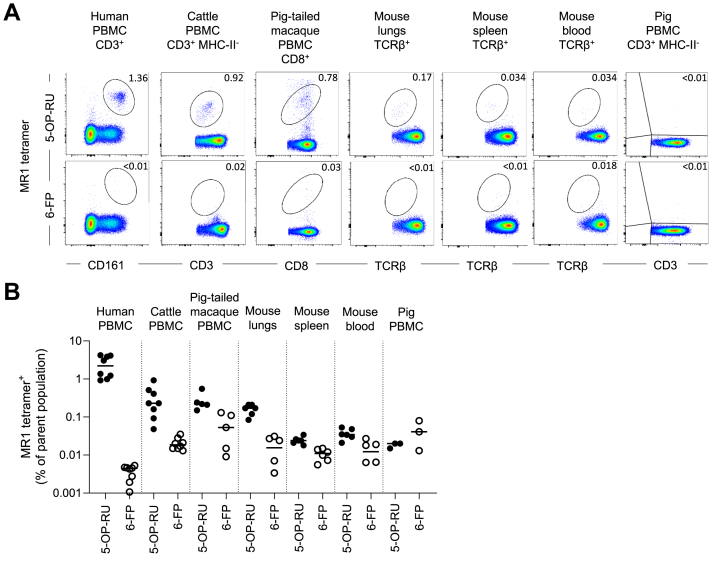
**Identification of MAIT cells in multiple species using species-specific MR1–5-OP-RU and MR1–6-FP tetramers.***A*, representative flow cytometry plots from staining of human PBMC, cattle PBMC, pig-tailed macaque PBMC, mouse lung-, spleen- and blood-derived lymphocytes, and pig PBMC with MR1–5-OP-RU and MR1–6-FP tetramers of the corresponding species. *B*, frequencies of species-matched MR1–5-OP-RU and MR1–6-FP tetramer^+^ MAIT cells from human CD3^+^ PBMC (n = 4), cattle CD3^+^ PBMC (n = 4), pig-tailed macaque CD8^+^ PBMC (n = 5), mouse lungs, spleen, and whole blood-derived αβTCR^+^ lymphocytes (n = 6), and pig CD3^+^ PBMC (n = 3). Mean ± SEM is depicted. Experiments were performed once in pig-tailed macaque and mouse and twice in all other species. 5-OP-RU, 5-(2-oxopropylideneamino)-6-d-ribitylaminouracil; 6-FP, 6-formylpterin; MAIT, mucosal-associated invariant T; MR1, major histocompatibility complex-I related protein 1; PBMC, peripheral blood mononuclear cell; TCR, T cell receptor.

Next, we utilized species-matched human, cattle, pig-tailed macaque, mouse, and pig MR1–5-OP-RU and MR1–6-FP tetramers to identify MAIT cells in their respective species (Figs. 2, S3, and S4). All blood samples tested were from healthy humans or animals. Mice (C57BL/6) were sourced from a specific pathogen-free Biological Research Facility; pig-tailed macaques (*Macaca nemestrina*) were sourced from a facility where pathogen exposure cannot be ruled out; pigs (Landrace x Common Large White breed) and cattle (Holstein Friesian breed) were both from high health status farms. The frequencies of MR1–5-OP-RU tetramer^+^ cells varied between species and individuals. Humans had the largest population, with a mean of 2.47% of CD3^+^ T cells being MR1–5-OP-RU tetramer^+^ in peripheral blood mononuclear cells (PBMC) (0.004% for MR1–6-FP tetramer). This finding is comparable to the previously described mean of 3.1% of CD3^+^ T cells (62). Cattle, alongside all other species, had a smaller proportion of MAIT cells with a mean of 0.32% of total CD3^+^ T cells in PBMC (0.02% for MR1–6-FP tetramer). This frequency of syngeneic MR1–5-OP-RU tetramer^+^ MAIT cells was lower than the 0.6% of total CD3^+^ T cells previously identified by human MR1–5-OP-RU tetramers (19). Pig-tailed macaque MAIT cells were previously shown to be almost entirely CD8^+^ (64). Accordingly, to facilitate gating, we utilized CD8 as a co-marker for MAIT cells in this species, identifying 0.27% of the total CD3^+^ population in PBMC as MR1–5-OP-RU tetramer^+^ (0.06% for MR1–6-FP tetramer). This frequency is comparable to that identified by Juno *et al.* (64). Mice had the smallest population identified, with 0.036% of TCRβ^+^ T cells being MR1–5-OP-RU tetramer^+^ in blood (0.012% for MR1–6-FP tetramer), 0.024% of TCRβ^+^ T cells being MR1–5-OP-RU tetramer^+^ in spleen (0.01% for MR1–6-FP tetramer), and a larger frequency of 0.16% of TCRβ^+^ T cells (0.015% for MR1–6-FP tetramer) in the lungs, similar to as previously described (49, 63, 71). Curiously, no discernible populations of pig MR1–5-OP-RU or MR1–6-FP tetramer^+^ cells were identified in PBMC of the three pigs tested.

### Complex MR1 cross-species reactivity based on MR1–5-OP-RU and MR1–6-FP tetramer staining

**Figure 3:**
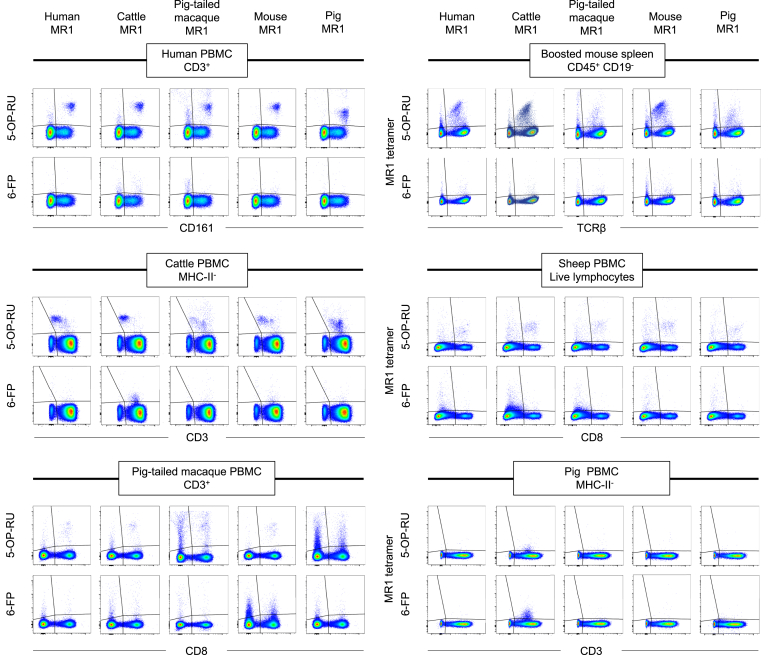
**Flow cytometry plots of species-mismatched MR1–5-OP-RU and MR1–6-FP tetramer staining.** Human, cattle, pig-tailed macaque, mouse, and pig MR1–5-OP-RU and MR1–6-FP tetramers were used to stain human PBMC, cattle PBMC, lymphocytes from MAIT cell boosted mouse spleen, pig-tailed macaque PBMC, sheep PBMC or pig PBMC. Representative final gating is shown for each species and each, MR1–5-OP-RU and MR1–6-FP tetramer. 5-OP-RU, 5-(2-oxopropylideneamino)-6-d-ribitylaminouracil; 6-FP, 6-formylpterin; MAIT, mucosal-associated invariant T; MR1, MHC-I related protein 1; PBMC, peripheral blood mononuclear cell.

To understand the extent to which MAIT cells have the capacity to cross-react with MR1 from different species, we undertook flow cytometric staining of human, cattle, pig-tailed macaque and pig PBMC with MR1–Ag tetramers from the different species. For mice, we opted to stain lymphocytes from spleen rather than blood. In naïve C57BL/6 mice, MAIT cell frequencies are comparable between blood and spleen (Fig. 2*B*) and mouse spleen MAIT cells mirror those in blood in frequency and phenotype (14, 72). However, absolute numbers of MAIT cells are much greater in spleen than in blood (Fig. S5*A*) and thus allow for the comparison between the whole panel of MR1 tetramers within the same animal. Nonetheless, it was difficult to robustly examine the small MAIT cell populations in naïve mouse spleen with MR1–Ag tetramers (Fig. 2). To facilitate comparative analysis of MR1–Ag tetramer staining, we included spleen from C57BL/6 mice that had been intravenously treated with 5-OP-RU in combination with the Toll-like receptor 9 agonist CpG, which leads to increased numbers of MAIT cells, including a 150-fold increase in the spleen (Fig. S5*A*), referred to as MAIT cell boosted mice (14, 15, 71). MAIT cells have only been identified genotypically in sheep (31), so we also examined PBMC from sheep sourced from high health status farms. To compare MR1–5-OP-RU and MR1–6-FP tetramer staining between species, cells from all species were stained with matching molarities of MR1–5-OP-RU and MR1–6-FP tetramers. In all cases, single live lymphocytes were sequentially gated as consistently as possible depending on antibody availability and subjected to a non-stringent final gate identifying MR1–5-OP-RU^+^ and MR1–6-FP tetramer^+^ cells. The parent gates of the MR1–5-OP-RU^+^ and MR1–6-FP tetramer^+^ cells involved CD3^+^ cells in the case of human, pig, cattle, and pig-tailed macaques, TCRβ^+^ cells for mice and CD8α^+^ cells for sheep, where no CD3-specific antibody was available (Figs. 3, S3, and S4).

**Figure 4:**
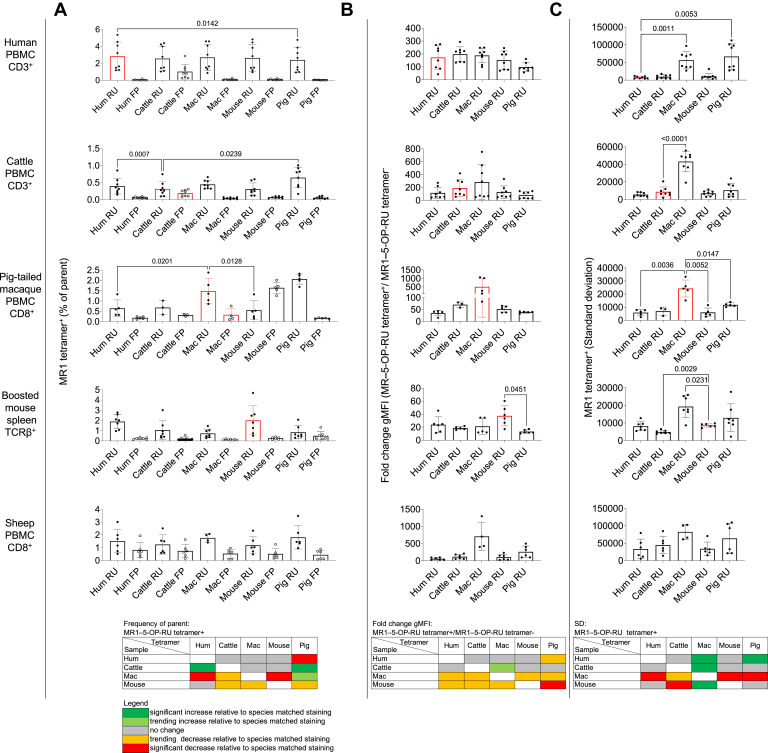
**Frequency and staining characteristics of MR1–5-OP-RU and MR1–6-FP tetramer**^**+**^**populations identified with species-matched and species-mismatched tetramer reagents.** Bar charts and heatmap summaries, displaying the following: *A*, frequencies of species-matched (*red histograms*) and species-mismatched (*black histograms*) MR1–5-OP-RU (RU, *black dots*) and MR1–6-FP (FP, *white dots*) tetramer^+^ cells in human CD3^+^ PBMC (n = 8), cattle CD3^+^ PBMC (n = 8), pig-tailed macaque CD8^+^ PBMC (n = 5), MAIT cell boosted mouse spleen (n = 7)–derived αβTCR^+^ lymphocytes, and sheep CD8^+^ PBMC (n = 6). Histograms depict mean frequency ± SEM with each individual datapoint shown. *B*, species-matched (*red* histograms) and species-mismatched (black histograms) MR1–5-OP-RU tetramer (RU) geometric mean fluorescence intensity (gMFI) fold change over the MR1–5-OP-RU tetramer^-^ population (background) and (*C*) SD of the MR1–5-OP-RU tetramer^+^ (RU) gMFI as described in (*A*). Differences between the frequencies obtained from species-matched and species-mismatched MR1–5-OP-RU staining were evaluated using a one-way ANOVA with Giesser-Greenhouse correction or, where there are missing values, a mixed effect model with repeated measures, followed by Dunnett’s multiple comparison test, comparing staining with species-matched MR1–5-OP-RU tetramer and all other species-mismatched MR1–5-OP-RU tetramers, with individual variance computed for each comparison. Only statistically significant differences (*p* value < 0.05) are indicated. Data are combined from either one (pig-tailed macaque), two (human, cattle, and sheep), or three (mouse) separate experiments. 5-OP-RU, 5-(2-oxopropylideneamino)-6-d-ribitylaminouracil; 6-FP, 6-formylpterin; PBMC, peripheral blood mononuclear cell; MAIT, mucosal-associated invariant T; MR1, MHC-I related protein 1; TCR, T cell receptor.

Flow cytometry plots (Fig. 3) and quantification of the frequencies of MR1–Ag tetramer^+^ cells (Fig. 4*A*) indicated that the majority of MR1–5-OP-RU tetramers reacted across species. There were few statistically significant differences between the findings obtained with species-matched *versus* mismatched MR1–Ag tetramers. There was a modest decrease in MAIT cell frequencies in human PBMC identified by the pig MR1-5-OP-RU tetramer. Fewer MAIT cells were also detected in pig-tailed macaque PBMC when using the human and mouse MR1-5-OP-RU tetramers. On the other hand, MAIT cell frequencies were higher in cattle PBMC when stained with the human and pig reagents and were increased compared to the species-specific cattle reagent (Fig. 4*A*, and Table 1). Though not significant, pig-tailed macaque, pig, and, to a lesser extent, cattle MR1–5-OP-RU tetramers identified a lower frequency (Fig. 4*A*, and Table 1) and absolute number (Fig. S5*B*) of MAIT cells in boosted mouse spleen than the mouse reagent. Consistent with 5-OP-RU antigen specificity, frequencies of MR1–6-FP tetramer^+^ T cells in PBMC from all species and from boosted mouse spleen were low (Figs. 3 and 4*A*, and Table 2).

**Table 1:** MR1–OP-RU tetramer staining characteristics

Cellular population	Parameter	Frequency MR1–5-OP-RU tetramer^+^					Fold change gMFI (MR1–5-OP-RU tetramer^+^/MR1–5-OP-RU tetramer^-^)					Standard deviation MR1–5-OP-RU tetramer^+^				
		Hum	Cattle	Mac	Mouse	Pig	Hum	Cattle	Mac	Mouse	Pig	Hum	Cattle	Mac	Mouse	Pig
Human PBMC CD3^+^	Value #	**8**	8	8	8	8	**8**	8	8	8	8	**8**	8	8	8	8
	Min	**1.19**	1.03	0.87	0.95	0.93	**43.12**	133.40	99.43	70.38	57.20	**4369**	4019	27042	3792	27155
	Max	**5.36**	4.39	4.59	4.82	4.77	**276.10**	293.50	249.60	245.90	161.60	**12067**	17711	96864	31675	112459
	Range	**4.17**	3.36	3.72	3.87	3.84	**233.00**	160.10	150.20	175.50	104.40	**7698**	13692	69822	27883	85304
	Mean	**2.84**	2.56	2.70	2.64	2.40	**173.60**	198.30	188.80	152.10	98.31	**7551**	9587	56347	10965	66967
	SD	**1.67**	1.47	1.56	1.56	1.45	**91.13**	57.14	55.96	70.59	33.14	**3043**	5262	24224	8645	36821
	SEM	**0.59**	0.52	0.55	0.55	0.51	**32.22**	20.20	19.78	24.96	11.72	**1076**	1860	8565	3056	13018
Cattle PBMC CD3^+^ MHC-II^-^	Value #	8	**8**	8	8	8	8	**8**	8	8	8	8	**8**	8	8	8
	Min	0.15	**0.10**	0.32	0.12	0.17	42.49	**55.30**	51.28	48.42	28.55	3230	**1828**	19175	2936	2936
	Max	0.86	**0.75**	0.66	0.60	1.00	271.70	**419.70**	752.10	331.70	143.60	8457	**20678**	55876	11417	23959
	Range	0.71	**0.65**	0.34	0.48	0.84	229.20	**364.40**	700.80	283.30	115.10	5227	**18850**	36701	8481	21023
	Mean	0.39	**0.31**	0.45	0.31	0.64	115.30	**190.30**	282.80	126.80	80.57	5521	**8726**	43260	6954	10527
	SD	0.23	**0.21**	0.13	0.18	0.30	87.03	**131.40**	268.00	96.33	53.46	2212	**5688**	11674	2895	7565
	SEM	0.08	**0.08**	0.04	0.06	0.10	30.77	**46.46**	94.74	34.06	18.90	782.2	**2011**	4127	1024	2675
Pig-tailed macaque PBMC CD8^+^	Value #	5	3	**5**	5	5	5	3	**5**	5	5	5	3	**5**	5	5
	Min	0.33	0.34	**0.86**	0.15	1.68	15.44	57.94	**82.84**	37.19	32.42	2439	3327	**15147**	2822	10079
	Max	1.32	1.02	**2.38**	1.32	2.33	43.94	79.66	**1062.00**	65.25	42.15	7869	9036	**33228**	11599	14027
	Range	1.00	0.68	**1.53**	1.17	0.64	28.50	21.72	**979.50**	28.07	9.72	5430	5709	**18081**	8777	3948
	Mean	0.64	0.68	**1.48**	0.55	2.06	34.70	70.39	**507.60**	51.72	38.59	5842	6942	**24281**	6001	11790
	SD	0.41	0.34	**0.61**	0.46	0.25	11.34	11.20	**489.20**	13.37	3.74	2328	3144	**6482**	3408	1479
	SEM	0.18	0.20	**0.27**	0.21	0.11	5.07	6.47	**218.80**	5.98	1.67	1041	1815	**2899**	1524	661.5
Boosted mouse spleen TCRβ^+^	Value #	7	7	7	**7**	7	6	5	5	**6**	6	7	7	7	**6**	7
	Min	0.96	0.32	0.26	**0.50**	0.30	11.19	15.69	11.81	**16.99**	10.25	5450	3832	8666	**7067**	2244
	Max	2.78	3.00	1.46	**4.70**	2.07	45.43	21.78	34.71	**60.45**	17.26	12789	6528	26049	**9821**	27708
	Range	1.82	2.69	1.19	**4.20**	1.77	34.23	6.088	22.90	**43.46**	7.00	7339	2696	17383	**2754**	25464
	Mean	1.89	1.05	0.73	**2.01**	0.84	23.83	18.61	21.32	**37.42**	13.19	8168	4876	19338	**8645**	12917
	SD	0.70	0.95	0.43	**1.43**	0.64	12.13	2.371	12.20	**15.43**	2.71	2577	936.9	6216	**1020**	7946
	SEM	0.26	0.36	0.16	**0.54**	0.24	4.95	1.06	5.46	**6.30**	1.11	974.1	354.1	2350	**416.5**	3003
Sheep PBMC CD8^+^	Value #	6	6	4	6	6	6	6	4	6	6	6	6	4	6	6
	Min	0.59	0.51	1.40	0.48	0.96	28.42	52.54	402.60	23.82	96.94	5554	14097	61283	15466	21252
	Max	2.51	3.01	2.07	2.33	3.49	98.10	207.60	1295.00	230.70	497.70	79506	84988	102671	70642	109832
	Range	1.92	2.50	0.67	1.86	2.53	69.68	155.10	892.80	206.90	400.80	73952	70891	41388	55176	88580
	Mean	1.25	1.51	1.76	1.18	1.81	59.69	118.30	707.70	103.50	260.90	33623	45045	82691	34004	64095
	SD	0.76	0.92	0.32	0.67	0.91	34.13	68.03	414.60	82.89	168.80	28384	24780	22017	19277	43093
	SEM	0.31	0.37	0.16	0.27	0.37	13.93	27.77	207.30	33.84	68.90	11588	10116	11008	7870	17593

The bold values highlight data from species matched staining, *e.g*., human samples stained with human tetramer, whilst regular font shows data from species mismatched font, e.g., human samples stained with cattle tetramer. In the matching figures, bar graphs of species-matched staining are in *red*, while bar graphs of species mismatched staining are in *black*.

**Table 2:** MR1–6-FP tetramer staining characteristics

Cellular population	Parameter	Frequency MR1–6-FP tetramer^+^					Fold change gMFI (MR1–6-FP tetramer^+^/MR1–6-FP tetramer^−^)				
		Hum	Cattle	Mac	Mouse	Pig	Hum	Cattle	Mac	Mouse	Pig
Human PBMC CD3^+^	Value #	**8**	8	8	8	8	**8**	8	8	8	8
	Min	**0.01**	0.25	0.04	0.03	0.01	**8.32**	8.48	6.96	13.81	6.70
	Max	**0.19**	2.86	0.28	0.21	0.06	**45.14**	28.22	20.03	64.83	89.15
	Range	**0.18**	2.61	0.24	0.18	0.06	**36.82**	19.74	13.08	51.02	82.46
	Mean	**0.05**	1.02	0.09	0.09	0.03	**26.03**	19.45	12.79	27.76	31.97
	SD	**0.06**	0.87	0.08	0.07	0.02	**13.78**	5.91	3.76	17.38	31.47
	SEM	**0.02**	0.31	0.03	0.02	0.01	**4.87**	2.09	1.33	6.15	11.13
Cattle PBMC CD3^+^ MHC-II^-^	Value #	8	**8**	8	8	8	8	**8**	8	8	8
	Min	0.04	**0.07**	0.01	0.04	0.02	23.60	**17.25**	24.35	35.19	26.13
	Max	0.10	**0.31**	0.07	0.10	0.11	145.30	**86.66**	177.60	110.60	81.45
	Range	0.06	**0.23**	0.06	0.06	0.09	121.70	**69.41**	153.20	75.44	55.32
	Mean	0.06	**0.19**	0.04	0.07	0.06	68.19	**45.96**	95.10	60.84	51.61
	SD	0.02	**0.09**	0.03	0.03	0.03	45.14	**26.21**	61.15	26.04	24.05
	SEM	0.01	**0.03**	0.01	0.01	0.01	15.96	**9.27**	21.62	9.21	8.50
Pig-tailed macaque PBMC CD8^+^	Value #	5	3	**4**	5	5	5	3	**4**	5	5
	Min	0.11	0.25	**0.11**	1.26	0.13	12.74	23.14	**32.86**	17.59	14.16
	Max	0.24	0.35	**0.75**	2.01	0.19	20.43	27.19	**121.10**	20.44	16.33
	Range	0.14	0.10	**0.64**	0.75	0.06	7.69	4.05	**88.28**	2.85	2.17
	Mean	0.18	0.31	**0.33**	1.64	0.16	15.14	25.76	**68.56**	18.79	15.10
	SD	0.06	0.05	**0.29**	0.27	0.02	3.06	2.27	**40.63**	1.46	0.90
	SEM	0.03	0.03	**0.15**	0.12	0.01	1.37	1.31	**20.31**	0.65	0.40
Boosted mouse spleen TCRβ^+^	Value #	7	7	7	**6**	7	6	5	5	**5**	5
	Min	0.10	0.12	0.08	**0.12**	0.18	9.16	7.883	8.26	**8.62**	8.30
	Max	0.38	0.57	0.21	**0.40**	1.33	19.64	13.57	15.33	**47.16**	10.88
	Range	0.28	0.45	0.13	**0.28**	1.14	10.48	5.691	7.07	**38.54**	2.58
	Mean	0.23	0.22	0.13	**0.26**	0.49	12	10.66	11.28	**18.40**	9.18
	SD	0.10	0.16	0.05	**0.11**	0.41	3.91	2.216	2.87	**16.29**	1.08
	SEM	0.04	0.06	0.02	**0.04**	0.15	1.596	0.991	1.28	**7.29**	0.48
Sheep PBMC CD8^+^	Value #	6	6	6	6	6	6	6	6	6	5
	Min	0.27	0.14	0.11	0.17	0.08	50.91	38.64	22.05	95.74	23.10
	Max	1.93	1.63	0.96	1.26	0.90	100.20	101.30	175.40	403.20	85.35
	Range	1.66	1.48	0.86	1.09	0.82	49.28	62.67	153.40	307.50	62.25
	Mean	0.81	0.74	0.53	0.51	0.44	65.66	61.28	92.80	245.90	46.42
	SD	0.58	0.51	0.35	0.40	0.36	19.87	23.12	63.28	106.70	25.04
	SEM	0.24	0.21	0.14	0.16	0.15	8.11	9.44	25.83	43.57	11.20

The bold values highlight data from species matched staining, e.g., human samples stained with human tetramer, whilst regular font shows data from species mismatched font, e.g., human samples stained with cattle tetramer. In the matching figures, bar graphs of species-matched staining are in red, while bar graphs of species mismatched staining are in black.

Though no sheep-specific MR1–Ag tetramer reagent was available, MR1–5-OP-RU tetramers from all species identified similar-sized populations of cells in sheep PBMC, amounting to a mean of 1.2 to 1.8% of CD8α^+^ T cells, the higher frequency being observed with pig MR1–5-OP-RU tetramer (Fig. 4*A*, and Table 1). This is higher than the previous qPCR-based estimate of <0.1% MAIT cells as a frequency of total sheep T cells (31). As with all other species assessed, the MR1–6-FP tetramer stained a much smaller population than the MR1–5-OP-RU reagent, regardless of the species of MR1–6-FP tetramer reagent (mean frequency of 0.44–0.81%) (Fig. 4*A*, and Table 2).

No discernible population of T cells was detected in pig PBMC with any of the species-mismatched MR1–5-OP-RU tetramers (Fig. 3). However, the pig MR1–5-OP-RU tetramer identified T cell populations across species with differing efficiencies. MAIT cell detection by pig MR1–5-OP-RU tetramers was comparable to that using species-specific MR1–5-OP-RU tetramers in pig-tailed macaques, MAIT cell boosted mice and sheep. However, pig MR1–5-OP-RU tetramers detected more MAIT cells in cattle and fewer in humans when compared with species-specific reagents (Figs. 3, and 4*A*).

### Differential apparent mean avidity of MR1–Ag tetramers in the staining of MAIT cells

To assess the level of MR1–Ag tetramer species-specificity and cross-reactivity in more detail, we next quantified the level of staining by MR1–Ag tetramers by comparing the signal (gMFI) in MR1–Ag tetramer^+^ cells relative to tetramer^-^ populations, expressed as a fold change in staining (Figs. 4*B*, and S6*A*). We use this measure as a proxy of the mean ‘relative avidity’ between MR1–Ag tetramers and MAIT TCRs in each species, mindful of assumptions, such as similar valence of MR1–Ag tetramer preparations. This echoes previous studies that established a correlation between MHC tetramer staining intensities and T cell avidity (73, 74), and we referred to this as the ‘apparent mean avidity’. MR1–5-OP-RU tetramer apparent mean avidity was highest in MAIT cells from pig-tailed macaque PBMC (fold change gMFI 607), followed by cattle (fold change gMFI 190) and human PBMC (fold change gMFI 173) and boosted mouse spleen (fold change gMFI 18 (Fig. 4*B*, and Table 1). The MR1–5-OP-RU tetramer apparent mean avidity was comparable between species-matched and species-mismatched reagents for all species tested. The exception was a reduced apparent mean avidity with pig MR1–5-OP-RU tetramer in boosted mouse spleen. Apparent mean avidities with pig-tailed macaque and cattle MR1–5-OP-RU tetramers also tended to be lower in mouse spleen, matching the pattern of MAIT cell frequencies identified with these reagents (Fig. 4*B*, and Table 1). In summary, there are subtle differences between the apparent mean TCR-MR1–5-OP-RU avidities within the MAIT cell populations between species, with the apparent mean avidity being highest for the pig-tailed macaque and lowest for the mouse MAIT–MR1 axis. Species-mismatched MR1–5-OP-RU tetramers can lead to a reduced mean avidity.

### Differential 5-OP-RU *versus* 6-FP specificity in MR1–Ag tetramer staining of MAIT cells

To understand the specificity for 5-OP-RU *versus* 6-FP in the context of species-matched and species-mismatched reagents in more detail, we determined the ratio of the apparent mean avidities of the MR1–5-OP-RU (Fig. 4*B*, and Table 1) and MR1–6-FP (Fig. S6*A*, and Table 2) tetramers by calculating the fold change gMFI of the MR1–5-OP-RU and the MR1–6-FP staining (Fig. S6*B*). Species-matched MR1–6-FP tetramers bound with much lower apparent mean avidities (pig-tailed macaque: 15.3-fold, human: 6.6-fold, cattle: 4.1-fold, boosted mouse spleen: 1.9-fold) than MR1–5-OP-RU tetramers (Fig. S6*B*). In fact, all MR1–Ag tetramer-staining combinations resulted in apparent avidity ratios above one and thus were more specific for 5-OP-RU *versus* 6-FP, except for sheep PBMC stained with human and mouse reagents (Fig. S6*B*). Another notable aspect of this analysis was that the specificity for the Ag 5-OP-RU *versus* 6-FP was most pronounced when staining human PBMC, with species-matched apparent avidity ratios of about seven-fold and even significantly higher increments with species-mismatched cattle and pig-tailed macaque reagents (Fig. S6*B*). In summary, despite some variation among species, with both species-matched and species-mismatched reagents, MR1–5-OP-RU tetramers showed stronger specificity in staining MAIT cells than MR1–6-FP tetramers. Given that MR1–6-FP tetramer^+^ populations were lower in frequency and possessed a lower mean apparent avidity than MR1–5-OP-RU tetramer^+^ populations, their precise specificity, CD8 dependency, and functional potential would require additional studies, as previously reported for human (59, 75, 76) and mouse MAIT cells (75, 76).

### Variability in the range of apparent MR1–Ag tetramer avidities for MAIT cells

To assess the level of MR1–Ag tetramer species-specificity and cross-reactivity in more detail, we also quantified the SD of the MR1–Ag tetramer fluorescence within the MR1–5-OP-RU tetramer^+^ populations (Fig. 4*C*, and Table 1). In principle, this acts as a surrogate for the range of apparent MAIT TCR-MR1–5-OP-RU tetramer avidities within each species, similar to previous studies evaluating CD1d–Ag tetramer avidities on NKT cells (77). Differences in the range of avidities between MR1–5-OP-RU tetramer and TCRs in MAIT cells from different species could reflect variations in the range of intrinsic affinities between MAIT TCRs and MR1–5-OP-RU. However, these experiments do not evaluate the impact of dose-dependencies of tetramer interactions as well as of CD8 co-receptors in the different species. The range of apparent avidities of species-matched MR1–5-OP-RU tetramer fluorescence was lowest in human PBMC (mean SD 7551), followed by boosted mouse spleen (mean SD 8645) and cattle PBMC (mean SD 8726) (Fig. 4*C*, and Table 1). Notably, pig-tailed macaque PBMC displayed a much greater SD of species-matched MR1–5-OP-RU tetramer fluorescence (mean SD 24281) (Fig. 4*C*, and Table 1). The range of apparent avidity of pig-tailed macaque MR1–5-OP-RU tetramer fluorescence was also significantly higher when staining boosted mouse spleen (2.23-fold), cattle PBMC (5.0-fold), and human PBMC (7.5-fold). Similarly, in human PBMC, the pig MR1–5-OP-RU tetramer fluorescence displayed a significantly higher SD (8.9 fold). Meanwhile, a significantly lower range in apparent MR1–5-OP-RU tetramer avidity was observed in pig-tailed macaques stained with all other species MR1–5-OP-RU tetramers (human: 4.2-fold; mouse: 4.0-fold; cattle: 3.5-fold; pig: 2.1-fold) (Fig. 4*C*, and Table 1). Accordingly, there is likely an intrinsic aspect unique to pig-tailed macaque MR1 that leads to varied binding by T cells in each species, rather than pig-tailed macaque MAIT TCRs having a greater range of intrinsic affinities for MR1 than found in other species.

### Further characterization of MAIT cells in pigs and sheep

**Figure 5:**
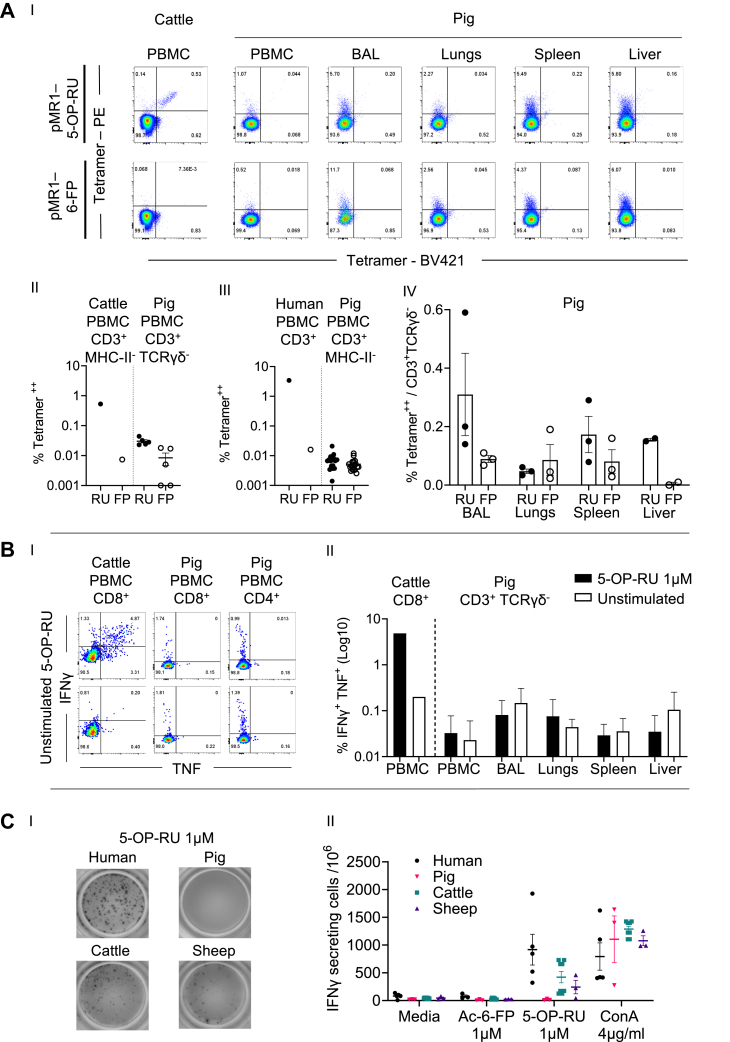
**Detailed characterization of MAIT cells in pigs and sheep.***A*, (I) representative flow cytometry plots of cells stained with pig MR1–5-OP-RU (pMR1–5-OP-RU) or MR1–6-FP (pMR1–6-FP) tetramers conjugated to PE or BV421 among CD3^+^ γδTCR^-^ pig PBMC, BAL, lungs spleen, and liver (and for comparison among CD3^+^ cattle PBMC), (II) the proportion of cells double positive for pig MR1–5-OP-RU (RU) or MR1–6-FP (FP) tetramers conjugated to PE or BV421 among CD3^+^ γδTCR^-^ T cells in pig PBMC (n = 5) (and for comparison among CD3^+^ MHC-II^-^ cattle PBMC (n = 1)), (III) the proportion of cells double positive for pig MR1–5-OP-RU (RU) or MR1–6-FP (FP) tetramers conjugated to PE or BV421 among CD3^+^ MHC-II^-^ pig PBMC (n = 18) (and for comparison among CD3^+^ human PBMC (n = 1)), and (IV) the proportion of cells double positive for pig MR1–5-OP-RU (RU) or MR1–6-FP (FP) tetramers conjugated to PE or BV421 among CD3^+^ γδTCR^−^ T cells in pig (n = 2 or 3) BAL, lungs, spleen, and liver lymphocytes. *B*, representative flow cytometry plots (I) and quantification of the frequency (II) of the IFNγ and TNF intracellular cytokine response in cattle CD8^+^ PBMC (n = 1) and CD3^+^ γδTCR^-^ pig (n = 3) PBMC, BAL, lungs, liver, and spleen lymphocytes, following stimulation with 1 μM 5-OP-RU for 6 h. Mean ± SEM are shown. *C*, representative images (I) and quantification (II) of secreted IFNγ assessed by ELISpot following coincubation of human (n = 3), pig (n = 3), cattle (n = 4), and sheep (n = 3) PBMC with medium, 1 μM Ac-6-FP, 1 μM 5-OP-RU, or 4 μg/ml ConA for 18 h. Mean SCF/10^6^ cells ± SEM are shown. Ac-6-FP, acetyl-6-FP; 5-OP-RU, 5-(2-oxopropylideneamino)-6-d-ribitylaminouracil; 6-FP, 6-formylpterin; MAIT, mucosal-associated invariant T; MHC, major histocompatibility complex; MR1, MHC-I related protein 1; TCR, T cell receptor; PBMC, peripheral blood mononuclear cell.

Previous quantification of MAIT cells in the Common Large White breed of pigs used in this study identified a low frequency of the canonical MAIT TCRα-chain in both PBMC (0.2% of TCRα chain) and tissues, including in lungs, liver, and spleen (55). Expression of the pig *MR1* gene was detected in PBMC and these tissues (34). However, in the absence of exogenous MR1 ligands, only intracellular but not cell surface pig MR1 protein was detected (34). Accordingly, our inability to identify pig MAIT cells with either species-matched or species-mismatched MR1–Ag tetramers was unexpected. The lack of detectable pig MAIT cells contrasts with the ability of the pig MR1–Ag tetramer to identify populations of cells in other species, suggesting that the pig MR1–5-OP-RU tetramer was functional (Figs. 3 and 4). Furthermore, most of the human PBMC that stained with human MR1–5-OP-RU tetramer co-stained with pig MR1–5-OP-RU tetramer (Fig. S7). Suspecting that the frequency of MR1–5-OP-RU tetramer^+^ cells might be low in pigs, cells were co-stained with pig MR1–Ag tetramers conjugated to two fluorophores to limit potential background staining (Fig. 5*A*, and S8*C*). As a control for this approach, we double stained cattle and human PBMC with pig MR1–Ag tetramers, which we previously determined could bind cattle and human MAIT cells as as well as the species-matched reagent (Figs. 3 and 4). Pig MR1–5-OP-RU tetramers independently fluorochrome tagged, identified a double positive–stained population in cattle (0.53% of CD3^+^ T cells) and human PBMC (3.43% of CD3^+^ T cells) while negligible double positive staining was observed with the MR1–6-FP reagent (Fig. 5*A*, I, II, III). The same MR1–5-OP-RU tetramer double staining method identified a low frequency population (0.03% CD3^+^ TCRγδ^-^ T cells and 0.007% CD3^+^ MHC-II^-^ cells) in pig PBMC. This result was consistent with our earlier findings and greater than the MR1–6-FP tetramer^+^ population of 0.008% CD3^+^ TCRγδ^-^ cells (Fig. 5*A*, I, II). In other species, such as cattle, humans, and mice, MAIT cells have previously been reported to be present in higher frequencies in tissues than in blood (3, 19, 63, 78). Thus, we applied the same MR1–5-OP-RU tetramer staining approach to pig tissues. We observed slightly greater mean frequencies of 0.31% (bronchoalveolar lavage), 0.04% (lungs), 0.15% (liver), and 0.17% (spleen) CD3^+^ TCRγδ^-^ cells (Fig. 5*A*, I, IV). However, varying levels of MR1–6-FP tetramer staining were also observed in these tissues (Fig. 5*A*, I, IV). Accordingly, using species-matched and species-mismatched MR1–5-OP-RU tetramer staining, only very low frequencies of MAIT cells were detected in PBMC (0.03%) compared with the 0.2% predicted by genomic analyses (55).

Next, we sought to assess the responsiveness of pig lymphocytes from PBMC and tissues to synthetic 5-OP-RU. Synthetic 5-OP-RU (1 μM) was used to stimulate the same PBMC and single cell suspensions from BAL, lungs, spleen, and liver previously stained with MR1–Ag tetramers. In intracellular cytokine staining assays, IFNγ/TNF double-positive cells were not identified beyond background (unstimulated controls) in any of the tissues. In contrast, cattle PBMC responded with a clear double positive population of cytokine-expressing T cells (Fig. 5*B*, I, II). We also used IFNγ ELISpot to quantify the number of T cells in pig PBMC responding to synthetic 5-OP-RU or Ac-6-FP. ELISpot results corroborated those of the intracellular cytokine staining, with no IFNγ-secreting cells detected above background. In contrast, under the same conditions, 5-OP-RU-specific IFNγ secretion was detected from human (mean 917 SFU/1e6 cells), cattle (mean 422 SFU/1e6 cells), and sheep (mean 243 SFU/1e6 cells) PBMCs (Fig. 5*C*, I, II). The frequency of IFNγ-secreting cells correlated with the proportion of MR1–Ag tetramer^+^ cells identified in each species (Fig. 4).

**Figure 6:**
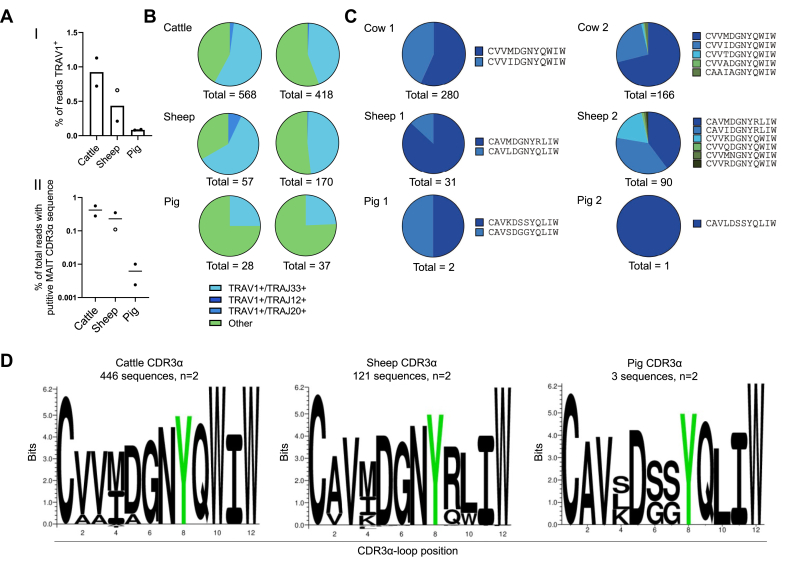
**Frequency and TCRα usage of putative MAIT cells in cattle, sheep and pig PBMC.***A*, frequencies of *TRAV1*^+^ (I) and putative MAIT TCR CDR3α sequence^*+*^ (II) of total *TRA* bulk sequenced cattle, sheep, and pig PBMC (n = 2). *B*, frequency of *TRAJ33*, *TRAJ12*, or *TRAJ20* gene segment usage among *TRAV1*^*+*^*TRA* sequences from cattle, sheep, and pig PBMC. *C*, relative frequency and sequence of putative MAIT TCR CDR3α-loops in each animal. *D*, sequence logo (Seq2Logo) of the amino acid weighting of the CDR3α-loops from putative MAIT TCRs in each species (note that the Y95, highlighted in *green*, is conserved between all species). MAIT, mucosal-associated invariant T; PBMC, peripheral blood mononuclear cell; TCR, T cell receptor.

To identify any putative MAIT cells in the pig, we performed unbiased bulk TRA sequencing in pig, alongside cattle and sheep PBMCs and determined the percentage of TRA chains expressing the *TRAV1* gene in each species. *TRAV* sequences were examined for putative MAIT TCR *TRAJ* gene usage (*TRAJ12/20/33*), CDR3α-loops of the expected length (12 amino acids), and sequence motifs (C(A/V)(A/V)xxxxYxxIW) (Fig. 6, and Table 3). *TRAV1* gene usage among TRA chains was higher in cattle (mean 0.925% of total TRA usage) and sheep (mean 0.435% of total TRA usage) than in pig (mean 0.085% of total TRA usage) PBMC (Fig. 6*A*, I). The frequency of the *TRAV1* gene usage among TRA chains also correlated with the frequency of T cells with putative MAIT TCR CDR3α-loop sequences among TRA chains (Fig. 6*A*, II). Among the putative *TRAV1*^*+*^ MAIT chains, *TRAJ33* was consistently the dominant *TRAJ* gene used (Fig. 6*B*). Based on expression of the canonical MAIT TCR CDR3α-loop, cattle had the largest population of MAIT cells with a mean of 0.47% of total TRA chains (Fig. 6*A*, II), followed by sheep with a mean of 0.23% of total TRA. The pig had a markedly smaller population of putative MAIT cells, with a mean of 0.004% of total pig TRA. This population identified in the pig was much lower in frequency than the previously reported 0.2% in pig PBMC (55). As expected, there was limited variation in the CDR3α-loops of MAIT TCRs identified within species and a high degree of sequence homology between species when considering the number of each CDR3α-loop sequence identified (Fig. 6*C*) and the amino acid weighting of aligned sequences (79) (Fig. 6*D*). Five distinct sequences out of the total 446 sequences were identified in cattle, all of which stemmed from *TRAV1-TRAJ33* rearrangements. In both animals, the CDR3α-loop sequences were dominated by CVVMDGNYQWIW and CVVIDGNYQWIW, which match the dominant sequences previously identified from cattle PBMC using human MR1–Ag tetramers (19). In sheep, seven CDR3α-loop MAIT TCR sequences were identified within the two animals, which were derived from *TRAV1-TRAJ33* rearrangements, except for a single sequence which was derived from a *TRAV1-TRAJ20* rearrangement. The CDR3α-loop sequences CAVMDGNYRLIW, CAVIDGNYRLIW, CVVKDGNYQWIW dominated in sheep PBMC, which agrees with a previous report (31). Three putative MAIT TCRs were identified from two pigs, each featuring a distinct CDR3α-loop sequence. Though present at a much lower frequency than in other species, the putative pig MAIT cell CDR3α-loops had close sequence homology to these species and were similar to the sequences obtained by the sequencing of *TRAV1-TRAJ33* transcripts previously reported (55).

**Table 3:** Putative MAIT TCR sequences

Animal	TRAV usage	TRAJ usage	CDR3α sequence	Number of transcripts	% of total putative MAIT cell sequences	% of total TCR CDR3α sequences	Combined % total TCR CDR3α sequences
Cow 1	TRAV1	TRAJ33	CVVMDGNYQWIW	159	56.8	0.32	0.56
	TRAV1	TRAJ33	CVVIDGNYQWIW	121	43.2	0.24	
Cow 2	TRAV1	TRAJ33	CVVMDGNYQWIW	181	79	0.31	0.39
	TRAV1	TRAJ33	CVVIDGNYQWIW	42	18.3	0.072	
	TRAV1	TRAJ33	CVVTDGNYQWIW	2	0.9	0.0034	
	TRAV1	TRAJ33	CVVADGNYQWIW	1	0.4	0.0017	
	TRAV1	TRAJ33	CAAIAGNYQWIW	3	1.3	0.0051	
Sheep 1	TRAV1	TRAJ33	CAVMDGNYRLIW	36	39.6	0.14	0.35
	TRAV1	TRAJ33	CAVIDGNYRLIW	34	37.4	0.13	
	TRAV1	TRAJ33	CVVKDGNYQWIW	17	18.7	0.066	
	TRAV1	TRAJ33	CVVQDGNYQWIW	1	1.1	0.0039	
	TRAV1	TRAJ33	CVVMNGNYQWIW	1	1.1	0.0039	
	TRAV1	TRAJ33	CVVRDGNYQWIW	1	1.1	0.0039	
	TRAV1	TRAJ20	CVVVLNNYKLTF	1	1.1	0.0039	
Sheep 2	TRAV1	TRAJ33	CAVMDGNYRLIW	27	87.1	0.097	0.11
	TRAV1	TRAJ33	CAVLDGNYQLIW	4	12.9	0.014	
Pig 1	TRAV1	TRAJ33	CAVKDSSYQLIW	1	50	0.0027	0.0055
	TRAV1	TRAJ33	CAVSDGGYQLIW	1	50	0.0027	
Pig 2	TRAV1	TRAJ33	CAVLDSSYQLIW	1	100	0.0024	0.0024

In summary, pig MR1–5-OP-RU tetramer identified a small population of MAIT cells in pigs. Unbiased bulk TCR sequencing of pig PBMC corroborated the low frequency of putative *TRAV1-TRAJ33*^+^ MAIT cells among *TRA* genes (0.0035%, average of two animals). This finding is much lower than previous data (0.2%) from Xiao *et al.* Synthetic 5-OP-RU–specific responses were not detected in pig T cells from PBMC or tissues, including those identified by MR1–5-OP-RU tetramers. Accordingly, our data suggest that MR1–5-OP-RU–specific MAIT cells are at the limits of phenotypic detection in all pigs tested. On the other hand, the same methods identified MAIT cells in cattle and sheep, corroborating MR1–Ag tetramer staining in these species and confirming that MAIT cells are an abundant population in sheep.

## Discussion

### Cross-species MR1–Ag tetramer reactivity

Most relevant to MR1-mediated xeno-reactivity are the TCR-exposed residues of the antigen-binding cleft of MR1, which differ across species. The key differentiating MR1 residues, mapped onto the crystal structure of human MR1 (Fig. S1, *B*–*F*), are mostly centered around the F′-pocket (indicated in Fig. S1*A*), whereas human MAIT TCRs dock over the human MR1 antigen-binding cleft orthogonally and centrally (8, 50, 51) away from the F′-pocket (Fig. S1*A*). This consensus footprint is also conserved when human MAIT TCRs interact with cattle MR1 (80, 81). Accordingly, almost all of the human MR1 residues, contacted by human MAIT TCRs *via* hydrogen bonds (Fig. S1*A*), are conserved between MR1 from humans and the other species (Fig. S1, *B*–*D*). One exception is MR1 R41Q in sheep and cattle (Fig. S1, *E* and *F*), with human MR1 R41 being a TCR contact in two (but not all) human MAIT TCR-MR1 crystal structures (51). The only human MR1 residues contacted by the MAIT TCR in all published human MR1-MAIT TCR structures are Y62 and Y152 (Fig. S1*A*). Both residues are conserved in pig-tailed macaque, mouse, pig, sheep, and cattle (Fig. S1, *A*–*G*) and Y152 is part of the ‘interaction triad’ that also includes the TCR Y95α residue and 5-OP-RU (52).

In line with the high level of MR1 and MAIT TCRα-chain conservation between species (2, 31, 32, 33, 35, 38, 40, 54, 55), xeno-reactivity between MAIT cells or MAIT TCRs and MR1 has been observed previously. For example, xeno-reactivity has been reported between mouse MAIT cells and human, cattle, and rat MR1 (9, 32), between human MAIT cells and rat, mouse and cattle MR1 (9, 32, 80, 81, 75, 76), between cattle MAIT cells and human MR1 (19), between bat MAIT cells and human MR1 (35), between thymic sheep MAIT cells and cattle MR1 (65), as well as thymic rat MAIT cells and mouse MR1 (65). However, the degree of cross-reactivity between human MR1 and MAIT cells from non-human primates appeared to vary (53, 54, 64), while macaque MR1 was able to bind to human MAIT cells (54). TCR xeno-reactivity can occur in an MR1-centric manner, independently of whether MR1 is loaded with exogenous antigen (32, 80, 81) or can be dependent on MR1 being loaded with exogenous antigen (9, 19, 35, 54, 64, 75, 76) and can sometimes be stronger than species-matched reactivity (35, 80).

Here, we utilized species-matched and species-mismatched MR1–Ag tetramer reagents on unfractionated cellular populations to determine the frequency, mean, and range of apparent MR1–Ag tetramer avidity and antigen specificity of the MAIT cell population. We identified the highest frequency of MAIT cells in humans, followed by cattle, sheep, pig-tailed macaques, and mice. The estimated apparent mean avidity of MR1–Ag tetramer binding was highest in pig-tailed macaque, followed by cattle, human, and then mice, suggesting that this key parameter of T cell activation is differentially tuned in different species, possibly to maintain antigen specificity *versus* T cell activation. This could, in part, be due to differential CD8 co-expression by the MAIT cell population between species, with CD8^+^ MAIT cells being dominant in humans (8, 49, 62, 82), cattle (19), and macaques (64) but not in mice (63) or pigs (55) and differential contributions from CD8-binding to MR1 to the apparent mean avidity depending on the species. Apart from studies in humans (50, 51, 59, 75), there are currently no affinity measurements of other species-matched MAIT TCRs and MR1 or of the MR1–CD8 interaction. Of note, however, affinities between TCR and peptide-MHC-I or peptide-MHC-II are >3-fold and >4-fold higher, respectively, in mice *versus* humans (83), potentially implying less dependence on co-receptor contributions to ligand binding and T cell activation in mice. Furthermore, the CD8-binding site is highly conserved in MR1 (59), and it has also been shown that human CD8 interacts with murine and human MHC-I molecules to a similar extent (84), and murine CD8 interacts to some extent with human MHC-I molecules (85). Accordingly, differential contributions from CD8-binding to MR1, as well as effects from cross-species interactions between CD8 and MR1, are less likely to drive observed differences in the apparent mean MR1 tetramer avidities.

We demonstrate a high level of cross-reactivity between MR1–5-OP-RU tetramers from evolutionarily distant species with populations of T cells identified in humans, sheep, cattle, pig-tailed macaques, and mice regardless of MR1–Ag tetramer species origin. In general, xeno-reactive MR1–5-OP-RU tetramer staining identified T cell populations that were similar in size and that co-stained by species-matched MR1–Ag tetramers, as tested for human and cattle MAIT cells. In addition, xeno-reactive MR1–5-OP-RU tetramer fluorescence intensity (apparent mean MR1–Ag tetramer avidity) and distribution of staining across positive T cells (apparent range of MR1–Ag tetramer avidity) were comparable to those observed with species-matched MR1–Ag tetramer staining. Furthermore, xeno-reactive MR1–Ag tetramer staining by MR1–6-FP tetramer was negligible or non-specific, indicating that xeno-reactive MR1–5-OP-RU tetramers identified antigen-specific T cell populations. There were some subtle differences in the cross-reactivity of MR1–Ag tetramers depending on the species. These differences resulted in the identification of an increased or reduced frequency of MR1–5-OP-RU tetramer^+^ MAIT cells, reduced apparent mean MR1–5-OP-RU tetramer avidity, and increased or reduced apparent range of MR1–5-OP-RU tetramer avidity. For instance, frequencies, absolute numbers, and fluorescent intensities were reduced when boosted mouse spleen was stained with pig-tailed macaque and pig relative to mouse MR1–5-OP-RU tetramers. This might be the result of a different charge within a TCR-exposed residue in mouse relative to pig and pig-tailed macaque MR1. Thus, substitution of the negatively charged glutamic acid (E159) in mouse MR1 to a polar uncharged glutamine in pig (E159Q), or a positively charged lysine (E159K) in pig-tailed macaque MR1 (Fig. S1, *B*–*D*) is likely to impair interaction of mouse MAIT TCRs with this region of the pig and pig-tailed macaque MR1 α2-domain. Furthermore, pig-tailed macaque MR1–5-OP-RU tetramer staining displayed the largest range of apparent avidity in pig-tailed macaques and all other species except in humans (second largest). This could possibly stem from the non-conserved TCR-exposed MR1 residue 151 that has previously been shown to mediate cross-species reactivity. Namely, residue 151 is a positively charged arginine in pig-tailed macaque MR1 *versus* a hydrophobic leucine in human MR1 or a polar uncharged glutamine in mouse, pig, sheep, and cattle MR1 (Fig. S1, *A*–*F*). Mutation of the residue L151 to Ala in human MR1 mediates MR1-reactivity by human MAIT TCRs in the absence of riboflavin-based antigens (86). Furthermore, L151 in human MR1 is a key determinant, preventing the activation of a mouse MAIT cell hybridoma by human MR1 (32). Moreover, partial humanization of cattle MR1 by mutating this residue to Q151L reduces the binding affinity to the human A-F7 MAIT TCR in the absence of riboflavin-based antigen from ∼30 μM to undetectable (80).

Reduced apparent mean MR1–5-OP-RU tetramer avidity and increased apparent range of MR1–5-OP-RU tetramer avidity typically impact the perceived MR1–Ag tetramer staining quality, leading to an underestimation of MAIT cell frequencies when identified by cross-species MR1–Ag tetramers. Therefore, we conclude that xeno-reactive MR1–Ag tetramers can be good proxies for the identification of MAIT cells in different species at the cell population level, but validation with species-matched MR1–Ag tetramers and/or TCR sequencing may be required to fully confirm MAIT cell identification. For instance, we previously found that 24 to 38% of CD8^+^ human MR1–5-OP-RU tetramer^+^ T cells in cattle did not express the canonical MAIT TCR, possibly due to nonspecific binding of MR1–5-OP-RU tetramer to non-MAIT cells alongside sorting impurities (19). Such a consideration would be particularly relevant when studying species with low MAIT cell frequencies, tissues, and following stimulation when apparent mean MR1–Ag tetramer binding avidity is reduced due to TCR downmodulation.

### Cross-species anti-MR1 antibody reactivity

It has previously been shown that changes in the MR1 protein sequence, particularly of residue Y152 located at the centre of the α2-domain of MR1 (Fig. S1*A*), impaired binding of the human MR1-specific mAb 26.5 to human MR1 (68) and it has been speculated that the mAb 26.5 binds more weakly to cattle MR1 in flow cytometry (32). We found that ELISA binding of mAb 26.5 to MR1–ligand was similar for human and mouse (64) but slightly weaker for pig, cattle (this study), and pig-tailed macaque MR1 (64). The ligand 5-OP-RU *versus* 6-FP had a limited impact on mAb 26.5 binding, although it appeared that in all cases, MR1–6-FP was slightly less well recognized than MR1–5-OP-RU. The mAb 8F2.F9, which recognizes a distinct epitope relative to the mAb 26.5 (69), also bound similarly to human and mouse MR1–ligand (64) and slightly weaker to MR1–ligand of pig-tailed macaque (64) and cattle (this study) in ELISA. Pig MR1–ligand (this study), however, was barely recognized and consistent with MR1 ligand-dependent binding; we could only detect binding to 6-FP– but not 5-OP-RU–loaded pig MR1. There was potentially also some very moderate MR1 ligand dependency with regards to mAb 8F2.F9 binding to other species MR1–ligand, but this varied between MR1 from different species. There are eight amino acid differences in the α1-/α2-domains of pig MR1 relative to mouse and human MR1, respectively (Fig. S1*G*). In particular, amino acid differences (mouse/human-pig) A/A55E (solvent exposed) and V/G18E (not solvent exposed) represent changes from small, non-polar residues to a larger, negatively charged glutamic acid which could potentially impact mAb 8F2.F9 binding (Fig. S1, *A*, *C*, and *D*). It is also possible that the polarity of the solvent-exposed ribityl chain of 5-OP-RU, which is absent in 6-FP, further impacts on mAb binding. Thus, changes in the A′-pocket may indirectly impact on epitope recognition in the area of the F′-pocket by the mAb 8F2.F9. The differential binding capacity of the mouse MR1-specific mAb 26.5 and the human-MR1-specific mAb 8F2.F9 to different species MR1–ligand have practical implications for their use in MR1-staining and blocking experiments in diverse species. For instance, mAb 26.5 does not block peripheral blood MAIT cell activation in bat (35) or in cattle (data not shown).

### MAIT cells in pigs

The level of MR1 cross-reactivity shown in this study, between species that have been separated by millions of years of evolution, is remarkable and suggests an important role of the MAIT–MR1 axis in immunity and/or homeostasis. By contrast, the loss of MR1 and MAIT cells in some species (40) implies they are evolutionarily dispensable. Moreover, the small number of MAIT cells in laboratory mouse strains suggests their potential redundancy (2, 63). Additionally, in this study, MR1–5-OP-RU–specific MAIT cells in pigs were at the limit of phenotypic detection, noting that further studies utilizing more animals and different breeds of pigs may be warranted to determine whether our finding applies more generally. Of the species examined here, pig MR1 diverges the most from human MR1 in the α1- and α2-domains (Fig. S1*H*), with six amino acid changes (A55E, K166R, I162V, L151Q, E159Q, and Q147W) in the MR1 region in which contacts are made by human MAIT TCRs (Fig. S1, *A*–*F*). Mouse MR1 has three amino acid changes (K173R, L151Q, and Q147L) in the MR1 region in which contacts are made by human MAIT TCRs (Fig. S1, *A*–*F*). Given the capacity of pig and mouse MR1–5-OP-RU tetramers to cross-react with MAIT cells in other species, the sequence differences between pig and mouse MR1 relative to MR1 of other species do not readily explain the lack of a discernible population of T cells in pigs stained with pig MR1–5-OP-RU tetramers and the low MAIT cell numbers in mice. It has previously been hypothesized that while MAIT cells are a prominent population in humans and cattle, they may be redundant, being substituted functionally by other cell types or even deleterious in other species (40). Indeed, iNKT and γδ T cell depletion in mice leads to increased MAIT cell numbers (87, 88), suggesting that iNKT and γδ T cells may compete for the same immunological niche as MAIT cells (87, 89). Consistent with this hypothesis, iNKT cells are identifiable in pigs (90), while functional iNKT cells could not be identified in ruminants (91, 92, 93), and iNKT cells are a much smaller population than MAIT cells in humans (94). There may also be other innate-like T cell subsets in mammals that have overlapping functional roles (40). For example, pigs, sheep, and cattle have a high frequency of other lipid-reactive T cells and γδ T cells (95, 96). There is high diversity in unconventional T cell populations between animals, and advances in the development of tools to study these populations in non-human species may lead to important discoveries in this area.

## Experimental procedures

### MR1 ligands

Ac-6-FP (Schircks Laboratories) was dissolved at 1 mM in water containing 17 mM NaOH. 5-OP-RU was synthesized as a 12.3 mM stock solution in d_6_-DMSO (quantified by NMR spectroscopy (97), as previously described (12, 98)).

### Human samples

Healthy donor PBMC samples stem from the University of Oxford biobank, with all samples screened for inappropriate infections, which may affect results. Samples were collected from healthy blood donors within the Oxford Gastrointestinal Cohort (16/YH/0247 and 21/YH/0206), approved by the Yorkshire and Humber Ethics committee. Written informed consent was obtained in all cases.

### Animal samples

All UK animal experiments were conducted within the limits of a United Kingdom Home office license under the Animal Scientific Procedures Act 1986 and were reviewed and approved by the Animal Welfare and Ethical Review Bodies of the institutes where the experiments were performed. All studies in this work abide by the Declaration of Helsinki principles.

#### Pigs

Pig blood and tissues were collected from 7- to 8-week-old Landrace x Common Large White pigs, obtained from a commercial high-health status herd (average weight of 15 kg) under procedure project license numbers P47CE0FF2 and PP2064443. Samples were collected as previously described (99). For TCR sequencing, adolescent or adult animals were sourced from the Roslin Institute (UK) and did not present with any clinical signs at the time of sampling.

#### Cattle

Healthy Holstein-Frisian cattle between 3 to 56 months of age were housed at the Edinburgh University farms (UK). Samples were collected as previously described under procedure project license numbers P803DD07A and PE854F3FC (19).

#### Sheep

Sheep blood samples for MR1–Ag tetramer staining and stimulation experiments were obtained from Charollais sheep of approximately 3 years of age from high-health status herds at the APHA (UK), procedure project license number PP1962684. For TCR sequencing, a black face and an indigenous cross breed sheep sourced from the Roslin Institute (UK) and Zambia, respectively, were used.

#### Pig-tailed macaques

Pig-tailed macaques (*M. nemestrina*) were housed in the Monash Animal Research Platform Gippsland Field Station, Australia. Adult male and female pig-tailed macaques (6–15 years old) were selected from a previously initiated vaccination study, where they were immunized with recombinant proteins formulated in monophosphoryl lipid A adjuvant. PBMCs were isolated and cryopreserved from whole blood by Ficoll gradient centrifugation as previously described (100). Macaque studies and related experimental procedures were approved by the Monash Animal Research Platform 1 Animal Ethics Committee (MARP-1 AEC, Ethics number 24539), Australia.

#### Mice

Mice were bred and housed in the Biological Research Facility of the Peter Doherty Institute for Infection and Immunity (Melbourne, VIC, Australia). Specific pathogen-free male C57BL/6 mice aged 8 to 9 weeks were used in experiments, after approval by The University of Melbourne Animal Ethics Committee (Ethics numbers 22017 and 23211). MAIT cell boosting of mice was done as described previously (14, 71). Briefly, C57BL/6 mice were intravenously injected at day 0 with synthetic 5-OP-RU antigen (190 μl at a concentration of 10 μM in PBS, diluted from DMSO stock solution at 5 mM) and Toll-like receptor 9 agonist CpG (10 nmol/mouse, 10 μl at 1 nM/μl in PBS) and at days 1, 2, and 4 with synthetic 5-OP-RU antigen (200 μl at a concentration of 10 μM in PBS, diluted from DMSO stock solution at 5 mM). CpG (B-class and P-class CpG) with the sequence:

5′T∗C∗G∗T∗C∗G∗T∗T∗T∗T∗G∗T∗C∗G∗T∗T∗T∗T∗G∗T∗C∗G∗T∗TT∗CG∗T∗CG∗A∗CG∗A∗T∗CG∗G∗C∗G∗CG∗C∗G∗C∗C∗G-3′ (∗phosphorothioate linkage) non-methylated cytosine–guanosine oligonucleotides was purchased from Integrated DNA Technologies.

### Tissue processing

Samples obtained from all livestock species were processed as previously described (19). Detailed protocols for the preparation of samples for flow cytometry from various mouse organs and blood were described elsewhere (58). In brief, for lungs, perfusion through the heart was performed with 10 ml cold PBS and lung single-cell suspensions were prepared by finely chopping the lungs, followed by collagenase (type III) digestion and passing through 70-μm cell strainers. Spleen was prepared by passing the tissue through 70-μm cell strainers. For blood, 350 to 750 μl were collected *via* a cardiac bleed into a 1 ml syringe containing 50 μl heparin. Red blood cells were lysed from lungs, blood, and spleen preparations with pre-warmed (37 °C) hypotonic buffer Tris-based amino chloride for 5 min (lungs: once or twice, blood: once) and 10 min (spleen: once), respectively. Prepared samples were kept on ice prior to staining for flow cytometry.

### Generation of MR1 monomers

*S. scrofa* (pig) and *B. taurus* (cattle) genes encoding β2m (pig GenBank ID: AB436775.1, cattle GenBank ID: BC118352.1) and the soluble portion of MR1 (pig GenBank ID: MH796644.1, cattle GenBank ID: FJ028657.1) codon optimized for *E. coli* expression were purchased (Life Technologies). The gene encoding the soluble portion of pig MR1 was modified to include a mutation to facilitate correct disulphide bond formation, C262S; G262 of cattle was not mutated. A C-terminal cysteine for biotinylation using Maleimide-PEG2 biotin (Thermo Fisher Scientific), C272, was introduced in the genes encoding the soluble portions of both pig and cattle MR1.

Genes encoding pig MR1 and β2m, as well as cattle MR1 and β2m were expressed separately in BL21 *E. coli*, and inclusion body protein was prepared and solubilized in 8 M urea, 20 mM Tris–HCl (pH 8), 0.5 mM Na-EDTA, and 1 mM DTT. MR1 (56 mg per 400 ml folding buffer) and β2m (28 mg per 400 ml folding buffer) were folded in the presence of 6-FP (2 ml per 400 ml folding buffer of a 5 mM solution of powder from Schircks Laboratories, dissolved in water, supplemented with 17 mM NaOH) or 5-amino-6-d-ribitylaminouracil [105 μl per 400 ml folding buffer of a 100 mM solution in DMSO generated in house (98)] and methylglyoxal (637 μl per 400 ml folding buffer of a 40% solution from Sigma-Aldrich), respectively by limiting dilution in a buffer adjusted to pH 8 to 8.5, containing 5 M urea, 100 mM Tris, 2 mM Na-EDTA, 400 mM L-arginine–HCl, 0.5 mM oxidized glutathione, 5 mM reduced glutathione, PMSF, and pepstatin A and dialyzed in 10 mM Tris before fast protein liquid chromatography purification by sequential DEAE anion exchange, gel filtration, and Mono-Q anion exchange chromatography. Purified protein was then reduced with 25 mM DTT for 15 min before buffer exchange into PBS using a PD-10 column (GE Healthcare). The cysteine-tagged MR1–5-OP-RU and MR1–6-FP monomers were biotinylated with Maleimide-PEG2 biotin (Thermo Fisher Scientific) with a 30:1 M ratio of biotin reagent to protein at 4 °C for 16 h in the dark and subjected to Mono-Q anion exchange chromatography to eliminate free biotin and isolate biotinylated MR1–5-OP-RU and MR1–6-FP monomers. Biotinylated MR1–5-OP-RU and MR1–6-FP monomers were stored at −80 °C.

### Tetramerization of MR1 monomers

For tetramerization, aliquots of biotinylated MR1 monomers were thawed out and tetramerized with streptavidin conjugated to PE (BD Biosciences) or, for tetramer co-staining, conjugated to Brilliant Violet 421 (BV421; Invitrogen), at an 1.8:1 M ratio of biotinylated MR1–ligand to streptavidin–fluorochrome to a final concentration of 3.2141 μM of biotinylated MR1–ligand monomer. A 10th of the volume of streptavidin fluorochrome conjugate was added to biotinylated MR1 monomer at 10-min intervals over 100 min, ensuring maximum occupancy of each streptavidin flurochrome conjugate with biotinylated MR1.

### Surface staining and flow cytometry

Cryopreserved cells from human, pig, cattle, sheep were thawed in RPMI media containing 10% fetal calf serum (FCS) and 1% L-glutamine/penicillin/streptomycin, and 2 × 10^6^ cells/well were seeded into a 96-well plate. Cells were stained with either MR1–5-OP-RU or MR1–6-FP tetramer at 1:400 (1:400 of 3.2141 μM biotinylated MR1-ligand monomer, *i.e.*, 8.035 nM) in PBS/2% FCS for 40 min at room temperature. Cells were then pelleted by centrifugation prior to staining with LIVE/DEAD Fixable Near-IR viability dye (Thermo Fisher Scientific) and species-matched antibody staining panels for MAIT cell identification (Table 4) in PBS/2% FCS for 30 min at 4 °C before acquisition on a MACSQuant Analyzer 10.

**Table 4:** List of antibodies

Species	Antigen	Clone	Isotype	Labeling strategy	Fluorochrome	Source of primary antibody	Staining concentration
Cattle	CD3	MM1A	IgG1	Secondary antibody	AF488	In-house	1:1000
	MHC-II	ILA21	IgG2a	Secondary antibody	AF647	In-house	1:1000
	CD8	ILA51	IgG1	Biotin-streptavidin	BV-421	In-house	1:500
	IFNγ	CC302	IgG1	Directly conjugated	AF647	Bio-Rad Laboratories	1:200
	TNF	CC327	IgG2b	Directly conjugated	AF488	Bio-Rad Laboratories	1:200
Human	CD3	BW264/56	IgG2a	Directly conjugated	Vioblue	Miltenyi Biotec	1:50
	CD161	191B8	IgG2b	Directly conjugated	APC	Miltenyi Biotec	1:50
Sheep	CD8	CC63	IgG2a	Secondary antibody	PercPcy5.5	In-house	1:1000
Pig	CD3	BB23-8E6-8C8	IgG2a	Directly conjugated	APC	BD Biosciences	1:100
	SLAll	2E9/13	IgG2b	Directly conjugated	FITC	Bio-Rad Laboratories	1:200
	CD3	BB23-8E6-8C8	IgG2a	Directly conjugated	PE-Cy7	BD Biosciences	1:100
	CD4	74-12-4	IgG2b	Directly conjugated	PerCP-Cy5.5	BD Biosciences	1:50
	CD8α	76-2-11	IgG2a	Directly conjugated	FITC	BD Biosciences	1:100
	TNF	MAb11	IgG2	Directly conjugated	BV421	BioLegend	1:200
	IFNγ	P2G10	IgG3	Directly conjugated	PE	BD Biosciences	1:600
	TCR1 delta	PGBL22A	IgG1	Secondary antibody	APC	Kingfisher	1:400
Mouse	CD44	IM7	IgG2b	Directly conjugated	BUV737	BD Biosciences	1:200
	CD45.2	104	Mouse SJL IgG2a, κ	Directly conjugated	FITC	BD Biosciences	1:200
	CD19	1D3	Rat IgG2a, κ	Directly conjugated	PerCPcy5.5	BD Biosciences	1:200
	TCRβ	H57-597	Armenian hamster/IgG	Directly conjugated	APC	BD Biosciences	1:200
Pig-tailed macaque	Human CD3	SP34-2	IgG1, λ	Directly conjugated	AF700	BD Biosciences	1:200
	Human CD8α	RPA-T8	IgG1, κ	Directly conjugated	BV650	BioLegend	1:200
	Human CD20	2H7	IgG2b, κ	Directly conjugated	BV510	BioLegend	1:200

Cryopreserved cells from pig-tailed macaques were thawed in RPMI media containing 10% FCS and 1% L-glutamine/penicillin/streptomycin, and 2 × 10^6^ cells were seeded into FACS tubes. Cells were stained with LIVE/DEAD Fixable Green viability dye (Thermo Fisher Scientific) followed by staining with either MR1–5-OP-RU or MR1–6-FP tetramer at 1:400 (1:400 of 3.2141 μM biotinylated MR1-ligand monomer, *i.e.*, 8.035 nM) in PBS/2% FCS for 5 to 10 min at room temperature. A mixture of surface mAbs (specific to human but cross-reactive with macaque) (Table 4) was then added and incubated with cells for 30 min at 4 °C before acquisition on an LSRFortessa (BD Biosciences) flow cytometer.

Mouse lymphocytes were used upon preparation from lungs, spleen, and blood. Two hundred microliters of blood from naïve mice, 30 to 70 μl of blood from MAIT cell boosted mice, one-eighth of spleen from naïve or MAIT cell boosted mice, and 1/10th of lungs from naïve mice were stained with Fixable Viability Dye eFluor 780 (Thermo Fisher Scientific) for 15 min on ice, followed by staining with surface mAbs (Table 4) and MR1–5-OP-RU or MR1–6-FP tetramer at 1:400 (1:400 of 3.2141 μM biotinylated MR1-ligand monomer, *i.e.*, 8.035 nM) for data in Figures 3 and 4 in PBS/2% FCS for 20 min at room temperature. Samples were filtered (40-μm cell strainer) and resuspended in PBS/2% FCS before acquisition on an LSRFortessa (BD Biosciences) flow cytometer. To determine absolute cell numbers, 123 count eBeads Counting Beads (Invitrogen) were diluted 1:4, counted using a hemocytometer, and 30 to 50 μl added to each sample prior to flow cytometric acquisition.

### Antigen stimulation of pig and cattle lymphocytes assessed by intracellular cytokine staining

Both Ac-6-FP and 5-OP-RU were diluted in RPMI containing 10% FCS to the required concentration immediately before use (DMSO content was less than 0.1% in the final media). Cells were co-incubated with 1 μM 5-OP-RU or 1 μM Ac-6-FP for 6 h with Brefeldin A (BioLegend) added for the final 4 h. Following stimulation, cells were stained for surface markers (Table 4) and near-IR fixable Live/Dead stain (Invitrogen) prior to fixation/permeabilization (BD Biosciences) and intracellular staining with antibodies (Table 4) for 25 min at 4 °C. Cells were then washed and resuspended in PBS for flow cytometer acquisition on a MACSQuant Analyzer 10.

### Antigen stimulation of cattle, pig, human, and sheep lymphocytes assessed by ELISpot

Frequencies of IFNγ-secreting cells were determined by ELISpot IFNγ-assay. MultiScreen-HA ELISPOT plates (Merck Millipore) were coated with primary anti-IFNγ pig clone P2G10 (BD Pharmingen 0.5 μg/ml), human clone 1-D1K (Mabtech-34 5 μg/ml), or cattle and sheep clone cc330 (Serotec, 2 μg/ml) and incubated at 4 °C overnight. Plates were washed and blocked with complete media for 2 h. Wells were seeded with 2.5 × 10^5^ human, cattle, pig, or sheep PBMC and stimulated with either 1 μM 5-OP-RU, 1 μM Ac-6-FP, 4 μg/ml ConA (Sigma-Aldrich), or media control. Following overnight incubation at 37 °C, plates were washed with PBS containing 0.05% Tween 20 and co-incubated with secondary biotinylated IFNγ detection Abs (Pig clone P2C11 (BD Pharmingen 0.5 μg/ml), human clone 7-B6-1 (Mabtech 0.25 μg/ml), cattle and sheep clone cc302 (Serotec 2 μg/ml)) for 2 h at room temperature. Plates were washed a further five times, and streptavidin–alkaline phosphatase (Invitrogen) was added for 1 h. Spots were visualized using an alkaline phosphatase substrate kit (Bio-Rad), and the reaction stopped using water. Immunospots were enumerated using the AID ELISPOT reader (AID Autoimmun Diagnostika). Results were expressed as the total number of IFNγ-producing cells per 10^6^ input PBMC after subtracting the average number of IFNγ^+^ cells in medium control wells.

### TCR sequencing

RNA was extracted from PBMC (Tri-reagent, Merck– used according to manufacturer’s protocol) and cDNA synthesized to append a universal primer-binding site at the 5′ end of transcripts using the SMART approach reported in (101). *TRA* chains were subsequently amplified using *TRAC*-specific reverse primers (Pig: GCATACACTGATGTTGTTAGATTTGG, sheep and cattle (ruminant): GGGCTTCTCAGCTGGTACAC) paired with a SMART-specific forward primer (AltUPM: GCA GTGGTATCAACGCAGAGT) with the Phusion HF PCR kit (New England Biolabs) and the following cycling conditions: 30 s at 98 °C, 30 cycles of (98 °C for 10 s, 65 °C for 30 s, 72 °C for 60 s), and a final extension period of 10 min at 72 °C. Following amplification, PCR products were quantified using Tapestation (Agilent), pooled to achieve equimolar quantities for the different samples, subjected to agarose gel electrophoresis and DNA bands of the correct size excised, and purified using AMPure XP beads (Beckman Coulter). Purified DNA was then sent to Edinburgh Genomics for Sequencing on the PacBio Sequel platform. Subsequent bioinformatics analysis was conducted using a combination of bespoke bioinformatics pipelines developed to cluster *TRA* reads based on in-house (The Roslin Institute, University of Edinburgh) livestock TCR sequence databases and manual annotation.

### Data analysis, including statistical analyses

All flow cytometry data were analyzed using FlowJo_v10.8.1 or v10.10.0. All statistical analyses were performed using Prism software version 8 (GraphPad). Data are presented as means with the SEM or SD.

## Data availability

All data are contained within the manuscript.

## Supporting information

This article contains supporting information (8, 51, 102, 103).

## Conflict of interest

J. Y. W. M., L. L., D. P. F., A. J. C., J. M., and S. B. G. E. are inventors on university owned patent rights (patent families WO/2015/149130 and WO/2014/005194) licensed for commercial use to Immudex and for non-profit use to the NIH Tetramer Core Facility. All other authors declare that they have no conflicts of interest with the contents of this article.
