# Supplementary material for: MAIT cell-MR1 reactivity is highly conserved across multiple divergent species
Source: J Biol Chem. 2024 May 3;300(6):107338. doi: 10.1016/j.jbc.2024.107338 (PMC11190491; doi:10.1016/j.jbc.2024.107338)
Supplement: Supporting Figures S1–S8 [file mmc1.pdf]

MAIT cell-MR1 reactivity is highly conserved across multiple divergent species

Matthew D Edmans[1,2#], Timothy K Connelley[3], Sophie Morgan[1], Troi J Pediongco[4], Siddharth Jayaraman[3], Jennifer A Juno[4], Bronwyn S Meehan[4], Phoebe M Dewar[4], Emmanuel A Maze[1], Eduard O Roos[1], Basudev Paudyal[1], Jeffrey YW Mak[5,6], Ligong Liu[5], David P Fairlie[5,6], Huimeng Wang[4,7], Alexandra J Corbett[4], James McCluskey[4], Lindert Benedictus[3,8*], Elma Tchilian[1*], Paul Klenerman[2*], Sidonia BG Eckle[4*#]

List of supporting material

Fig. S1 Comparative structural, sequence and phylogenetic analyses of MR1 in different species.
Fig. S2 Protein fold of cattle and pig MR1–5-OP-RU and MR1–6-FP monomers assessed in ELISA (isotype controls).
Fig. S3 Representative gating strategy for MR1–5-OP-RU and MR1–6-FP tetramer-based MAIT cell identification in human, cattle, sheep and pig-tailed macaque PBMC.
Fig. S4 Representative gating strategy for MR1–5-OP-RU and MR1–6-FP tetramer-based MAIT cell identification in mouse lungs, spleen and blood.
Fig. S5 Total numbers of MAIT cells in spleen and blood in naïve and in MAIT cell boosted mice.
Fig. S6 Fold change gMFI of the MR1–6-FP tetramer^{+} relative to the MR1–6-FP tetramer^{-} staining and fold change gMFI of the MR1–5-OP-RU tetramer^{+} to MR1–6-FP tetramer^{+} staining.
Fig. S7 Co-staining of human or cattle PBMC with species-matched and -mismatched MR1–5-OP-RU and MR1–6-FP tetramers.
Fig. S8 Representative gating strategy for pig and cattle MAIT cell identification by MR1–5-OP-RU tetramer double staining or 5-OP-RU stimulation and intracellular cytokine staining.

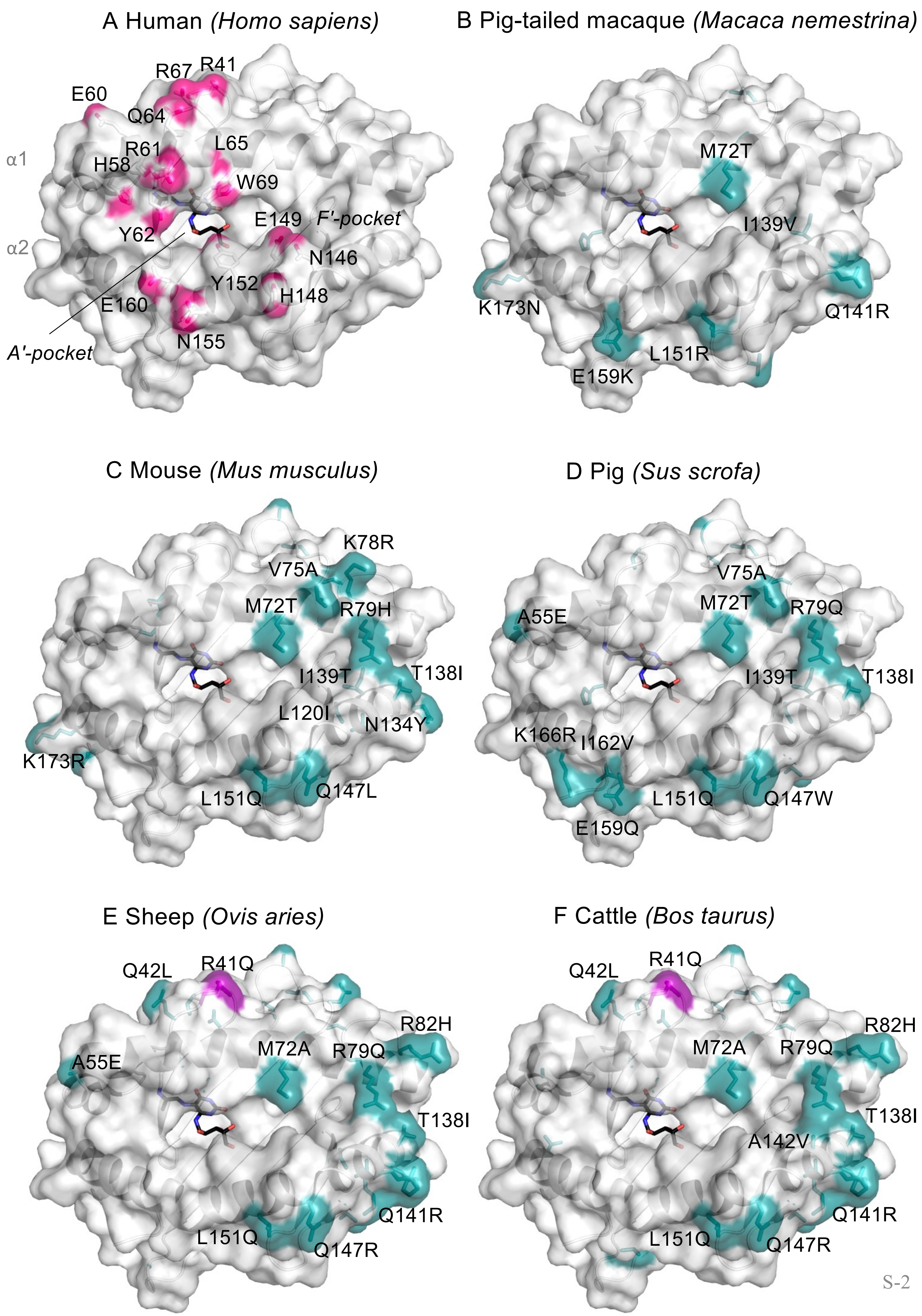
A Human (Homo sapiens)
R67
R41
E60
Q64
L65
R61
α1
H58
W69
E149
F'-pocket
Y62
α2
N146
Y152
H148
E160
N155
A'-pocket
B Pig-tailed macaque (Macaca nemestrina)
M72T
I139V
K173N
Q141R
L151R
E159K
C Mouse (Mus musculus)
K78R
V75A
M72T
R79H
I139T
T138I
L120I
N134Y
K173R
L151Q
Q147L
D Pig (Sus scrofa)
V75A
M72T
R79Q
A55E
I139T
T138I
K166R
I162V
L151Q
Q147W
E159Q
E Sheep (Ovis aries)
Q42L
R41Q
R82H
A55E
M72A
R79Q
T138I
Q141R
L151Q
Q147R
F Cattle (Bos taurus)
Q42L
R41Q
R82H
M72A
R79Q
T138I
A142V
Q141R
L151Q
Q147R

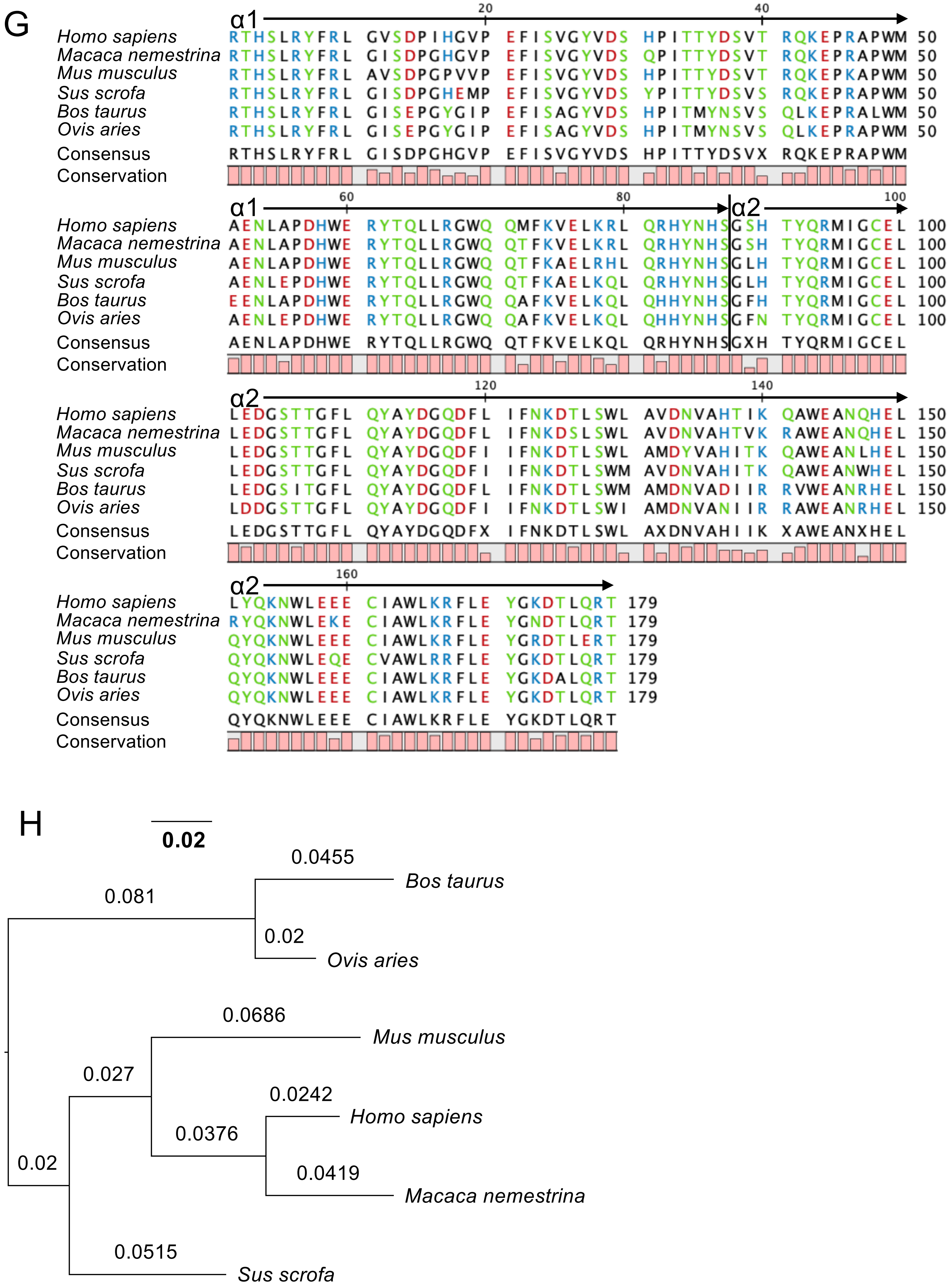
G
α1
20
40
Homo sapiens RTHSLRYFRL GVSDPIHGVP EFISVGYVDS HPITTYDSVT RQKEPRAPWM 50
Macaca nemestrina RTHSLRYFRL GISDPGHGVP EFISVGYVDS QPITTYDSVT RQKEPRAPWM 50
Mus musculus RTHSLRYFRL AVSDPGPVVP EFISVGYVDS HPITTYDSVT RQKEPKAPWM 50
Sus scrofa RTHSLRYFRL GISDPGHEMP EFISVGYVDS YPITTYDSVS RQKEPRAPWM 50
Bos taurus RTHSLRYFRL GISEPGYGIP EFISAGYVDS HPITMYNSVS QLKEPRALWM 50
Ovis aries RTHSLRYFRL GISEPGYGIP EFISAGYVDS HPITMYNSVS QLKEPRAPWM 50
Consensus RTHSLRYFRL GISDPGHGVP EFISVGYVDS HPITTYDSVX RQKEPRAPWM
Conservation
α1
α2
60
80
100
Homo sapiens AENLAPDHWE RYTQLLRGWQ QMFKVELKRL QRHYNHSGSH TYQRMIGCEL 100
Macaca nemestrina AENLAPDHWE RYTQLLRGWQ QTFKVELKRL QRHYNHSGSH TYQRMIGCEL 100
Mus musculus AENLAPDHWE RYTQLLRGWQ QTFKAELRHL QRHYNHSGLH TYQRMIGCEL 100
Sus scrofa AENLEPDHWE RYTQLLRGWQ QTFKAELKQL QRHYNHSGLH TYQRMIGCEL 100
Bos taurus EENLAPDHWE RYTQLLRGWQ QAFKVELKQL QHHYNHSGFH TYQRMIGCEL 100
Ovis aries AENLEPDHWE RYTQLLRGWQ QAFKVELKQL QHHYNHSGFN TYQRMIGCEL 100
Consensus AENLAPDHWE RYTQLLRGWQ QTFKVELKQL QRHYNHSGXH TYQRMIGCEL
Conservation
α2
120
140
Homo sapiens LEDGSTTGFL QYAYDGQDFL IFNKDTLSWL AVDNVAHTIK QAWEANQHEL 150
Macaca nemestrina LEDGSTTGFL QYAYDGQDFL IFNKDSLSWL AVDNVAHTVK RAWEANQHEL 150
Mus musculus LEDGSTTGFL QYAYDGQDFI IFNKDTLSWL AMDYVAHITK QAWEANLHEL 150
Sus scrofa LEDGSTTGFL QYAYDGQDFI IFNKDTLSWM AVDNVAHITK QAWEANWHEL 150
Bos taurus LEDGSITGFL QYAYDGQDFL IFNKDTLSWM AMDNVADIIR RVWEANRHEL 150
Ovis aries LDDGSTTGFL QYAYDGQDFI IFNKDTLSWI AMDNVANIIR RAWEANRHEL 150
Consensus LEDGSTTGFL QYAYDGQDFX IFNKDTLSWL AXDNVAHIIK XAWEANXHEL
Conservation
α2
160
Homo sapiens LYQKNWLEEE CIAWLKRFLE YGKDTLQRT 179
Macaca nemestrina RYQKNWLEKE CIAWLKRFLE YGNDTLQRT 179
Mus musculus QYQKNWLEEE CIAWLKRFLE YGRDTLERT 179
Sus scrofa QYQKNWLEQE CVAWLRRFLE YGKDTLQRT 179
Bos taurus QYQKNWLEEE CIAWLKRFLE YGKDALQRT 179
Ovis aries QYQKNWLEEE CIAWLKRFLE YGKDTLQRT 179
Consensus QYQKNWLEEE CIAWLKRFLE YGKDTLQRT
Conservation
H
0.02
0.0455
Bos taurus
0.081
0.02
Ovis aries
0.0686
Mus musculus
0.027
0.0242
Homo sapiens
0.0376
0.02
0.0419
Macaca nemestrina
0.0515
Sus scrofa

Fig. S1 Comparative structural, sequence and phylogenetic analyses of MR1 in different species. A-F) Cartoon and surface display (light grey) of the MR1 Ag-binding cleft and stick display of 5-OP-RU (black) based on the protein data bank (PDB) deposited crystal structure of the human A-F7 MAIT TCR in complex with human MR1–5-OP-RU (PDB ID: 4NQC, (8). **A**) MR1 atoms contacted in hydrogen bonds (there are no residues contacted via salt bridges) in any of available ternary crystal structures of human MAIT TCRs in complex with human MR1–5-OP-RU (PDB IDs: 4NQC (8); PDB IDs: 4PJ7, 4PJ8, 4PJ9, 4PJA, 4PJB, 4PJC, 4PJD (51)) are highlighted in magenta, residue side-chains displayed in stick format and residues labelled. All contacts include atoms of side-chains except for L65, where the main chain is contacted. The only MR1 residues contacted in all analyzed structures are Y62 and Y152. A'- and F'-pockets are indicated. **B-F**) Side-chains of residues that differ between human MR1 and MR1 of other species (as indicated) are highlighted in teal and residue side-chains displayed in stick format. Differing residues at the TCR-MR1 interphase are labelled as follows: human amino acid residue – residue number – other species amino acid residue. Except for R41 in sheep and cattle (E, F; highlighted in purple), all of the human MR1 residues contacted by human TCRs in hydrogen bonds are conserved between human and other MR1 species (B-D). R41 is a TCR contact in 2 crystal structures (PDB IDs: 4PJB, 4PJD (51). **A-F)** All figures were generated with the software The PyMOL Molecular Graphics System, Version 2.3.5 Schrödinger, LLC, upon contact analysis using PDBsum (102). **G)** Protein sequence alignment of the α1- and α2-domains (indicated) of MR1 from *Homo sapiens* (human, GenBank ID: AJ249778.1), *Macaca nemestrina* (pig-tailed macaque, NCBI Reference Sequence: XM_011746239.*1*), *Mus musculus* (mouse, GenBank ID: AF035672.1), *Sus scrofa* (pig, GenBank ID: MH796644.1), *Bos taurus* (cattle, GenBank ID: FJ028657.1) and *Ovis aries* (sheep, GenBank ID: FJ423039.1) using the Qiagen CLC Genomics Workbench 21.0. The consensus sequence and level of conservation are shown. Residues are coloured based on polarity, where hydrophobic residues are shown in black, hydrophilic residues in green, acidic residues in red and basic residues in blue colour. **H)** Phylogenetic tree inferred from the protein sequence alignment of the α1- and α2-domains of MR1 as per A using IQ-Tree 2 by maximum likelihood (103) and FigTree (Andrew Rambaut, Computer program distributed by the author, website: http://tree.bio.ed.ac.uk/software/figtree/) for visualization, indicating the branch length and scale.

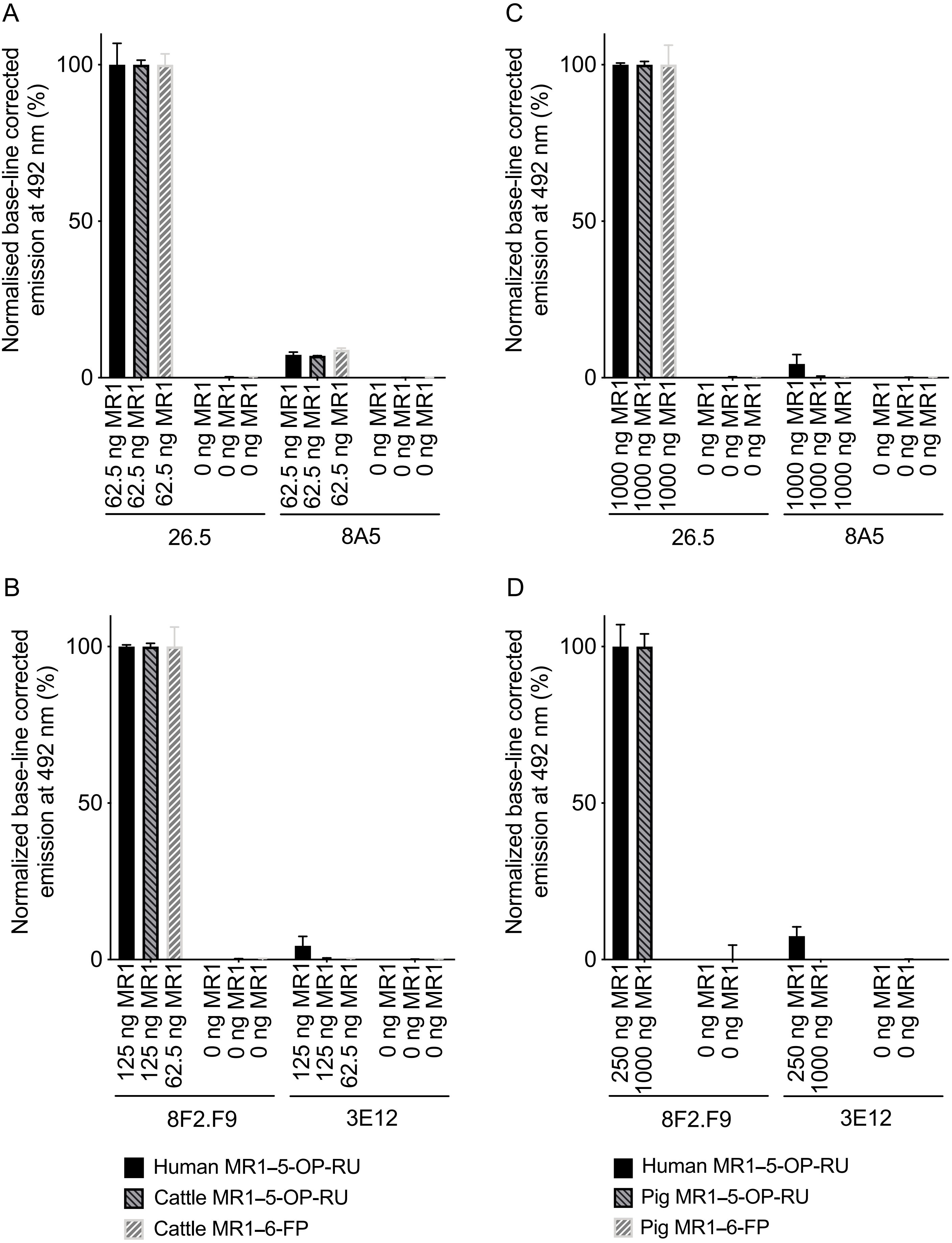

Fig. S2 Protein fold of cattle and pig MR1–5-OP-RU and MR1–6-FP monomers assessed in ELISA (isotype controls). Isotype control mAbs 8A5 (**A, C**) and 3E12 (**B, D**) of the assessment of the conformational integrity of biotinylated 5-OP-RU and 6-FP loaded cattle (**A, B**) and pig (**C, D**) MR1 molecules in comparison to biotinylated human MR1–5-OP-RU in ELISA with mAbs 26.5 (A, C) and 8F2.F9 (B, D). This data was generated as part of the data displayed in Fig. 1C.

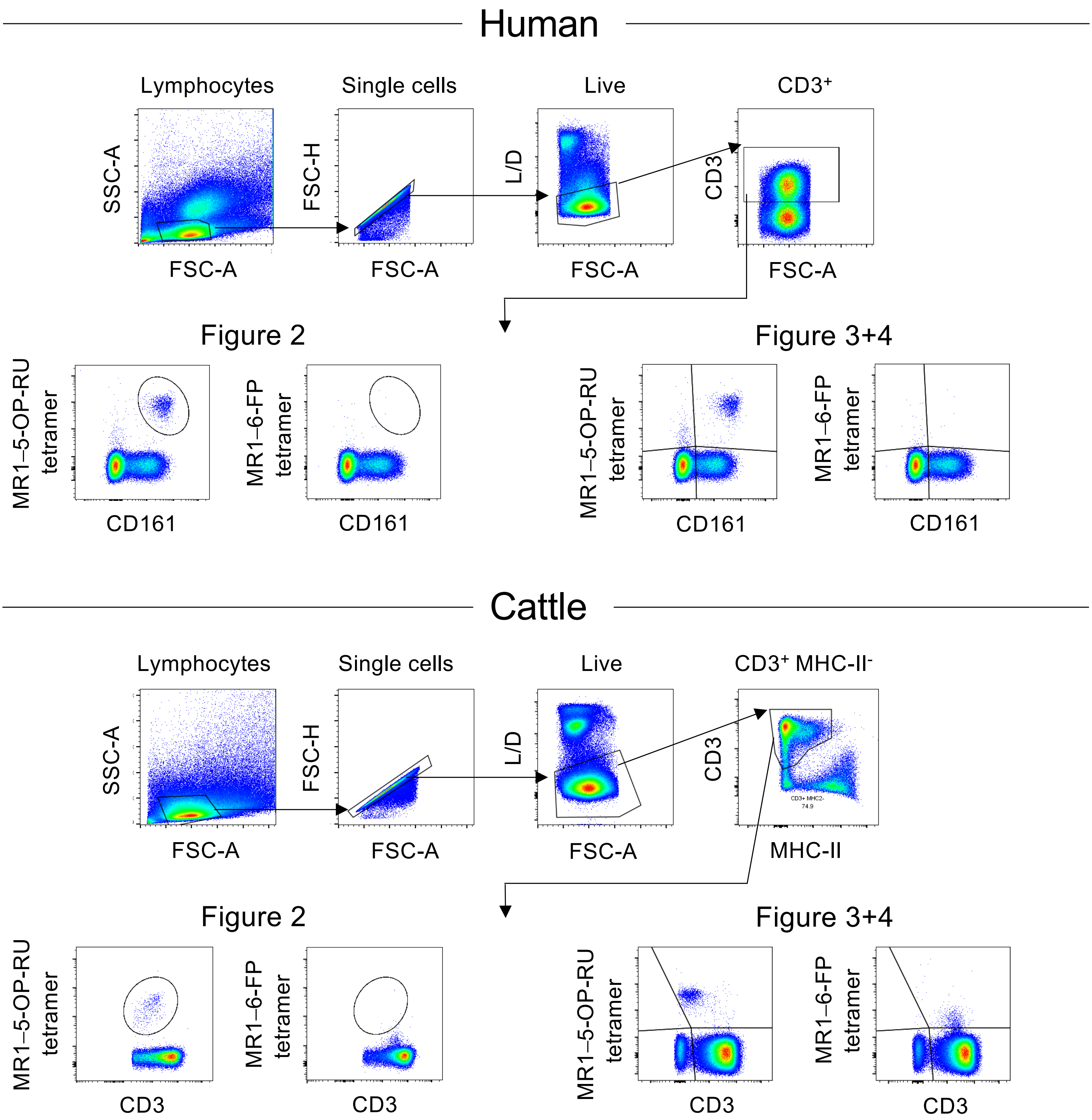

Human
Lymphocytes
Single cells
Live
CD3+
SSC-A
FSC-H
L/D
CD3
FSC-A
FSC-A
FSC-A
FSC-A
Figure 2
Figure 3+4
MR1–5-OP-RU tetramer
MR1–6-FP tetramer
MR1–5-OP-RU tetramer
MR1–6-FP tetramer
CD161
CD161
CD161
CD161
Cattle
Lymphocytes
Single cells
Live
CD3+ MHC-II-
SSC-A
FSC-H
L/D
CD3
FSC-A
FSC-A
FSC-A
MHC-II
Figure 2
Figure 3+4
MR1–5-OP-RU tetramer
MR1–6-FP tetramer
MR1–5-OP-RU tetramer
MR1–6-FP tetramer
CD3
CD3
CD3
CD3

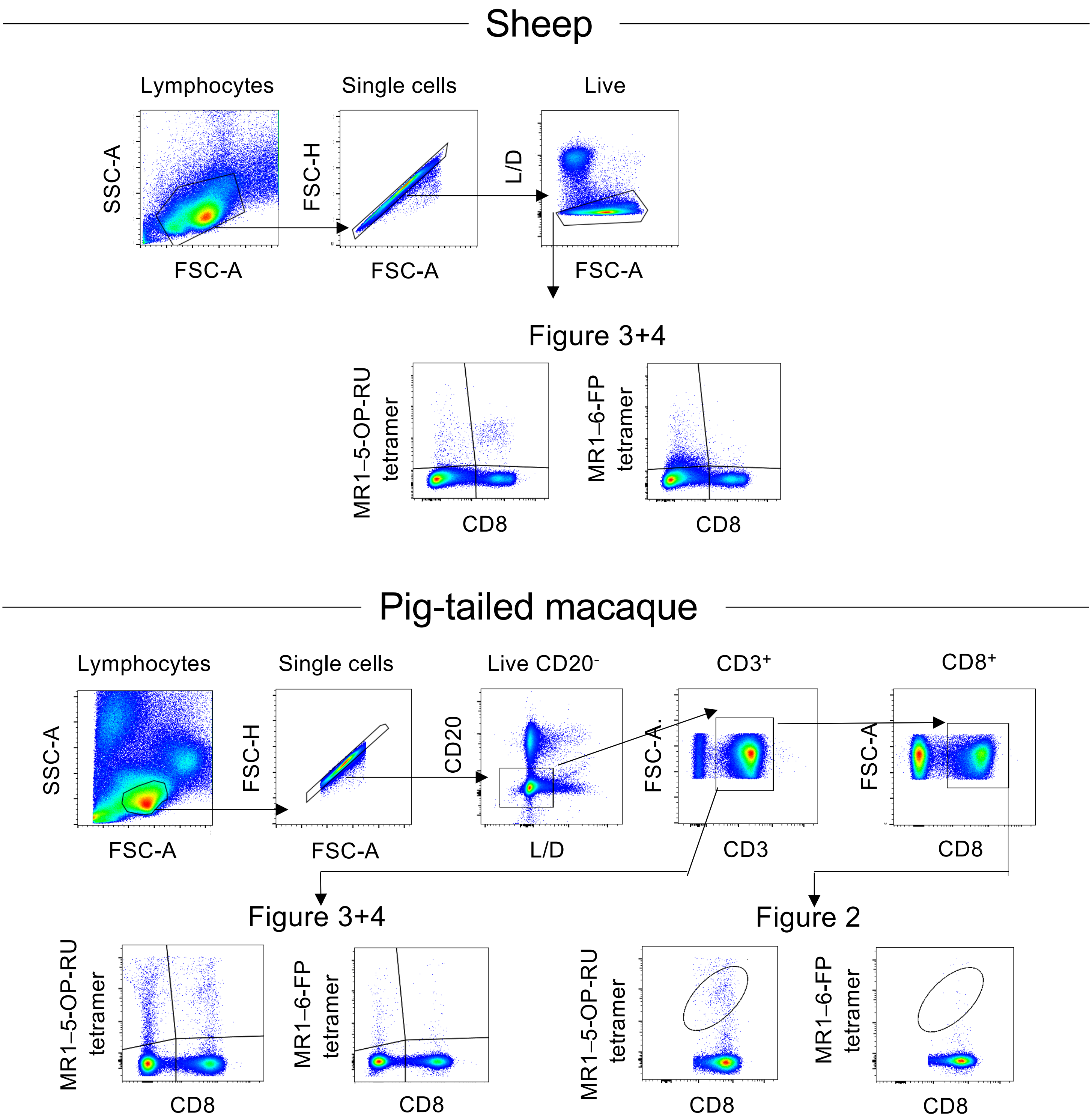

Fig. S3 Representative gating strategy for MR1–5-OP-RU and MR1–6-FP tetramer-based MAIT cell identification in human, cattle, sheep and pig-tailed macaque PBMC. Gating and staining strategy for each species as depicted in Fig. 2 (species-matched MR1–5-OP-RU and MR1–6-FP tetramer MAIT cell identification) and Fig. 3 (species-mismatched MR1–5-OP-RU and MR1–6-FP tetramer MAIT cell identification) are shown.

Mouse lungs

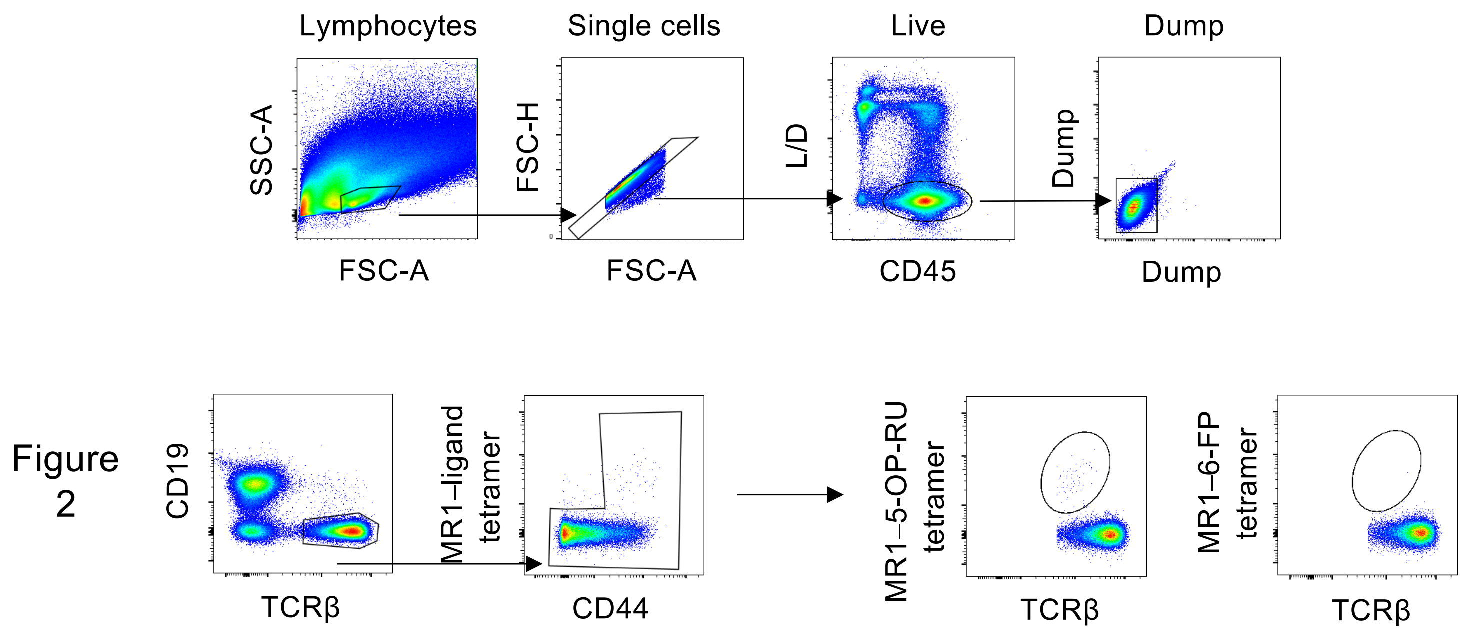

Mouse spleen

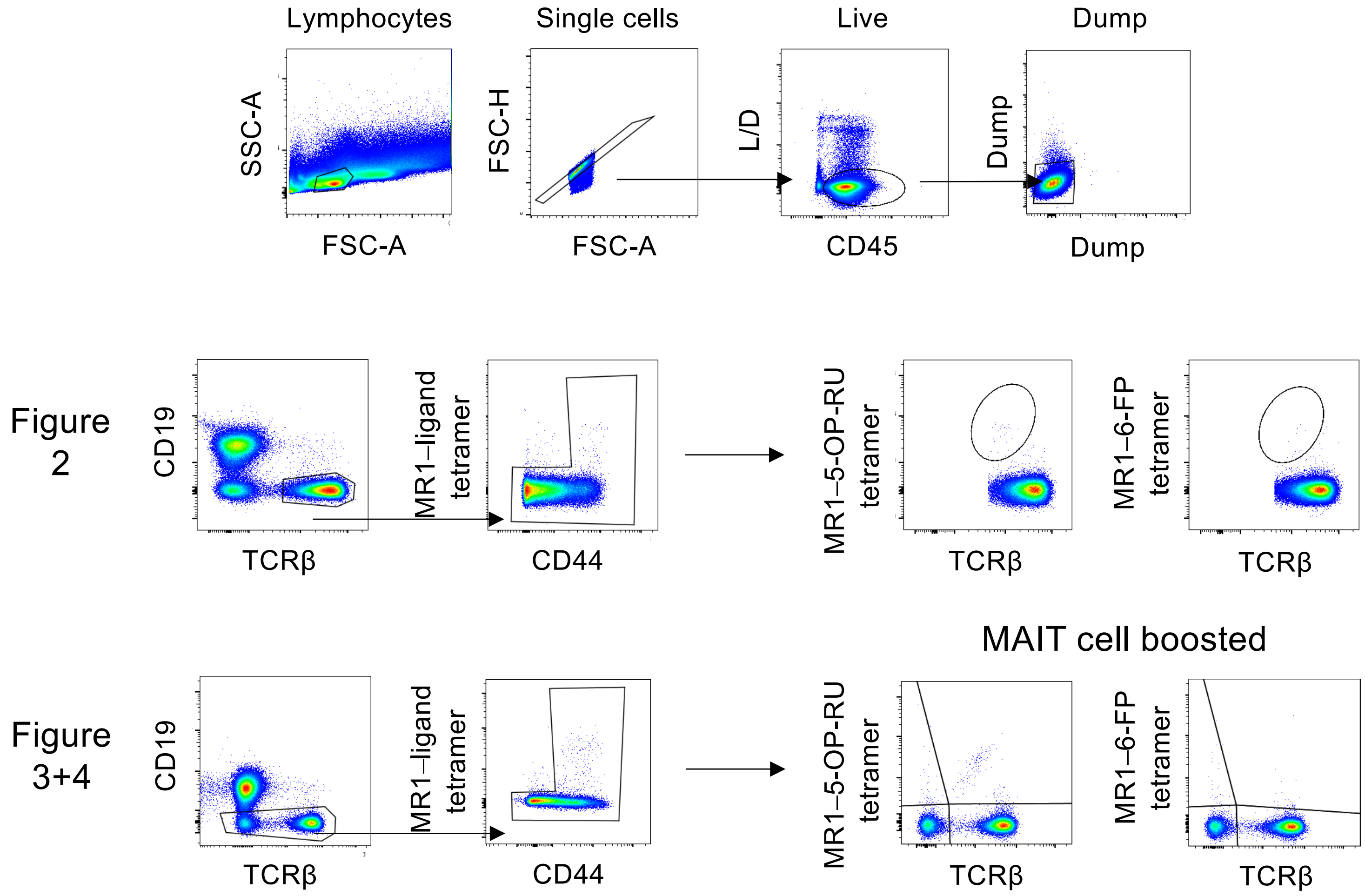

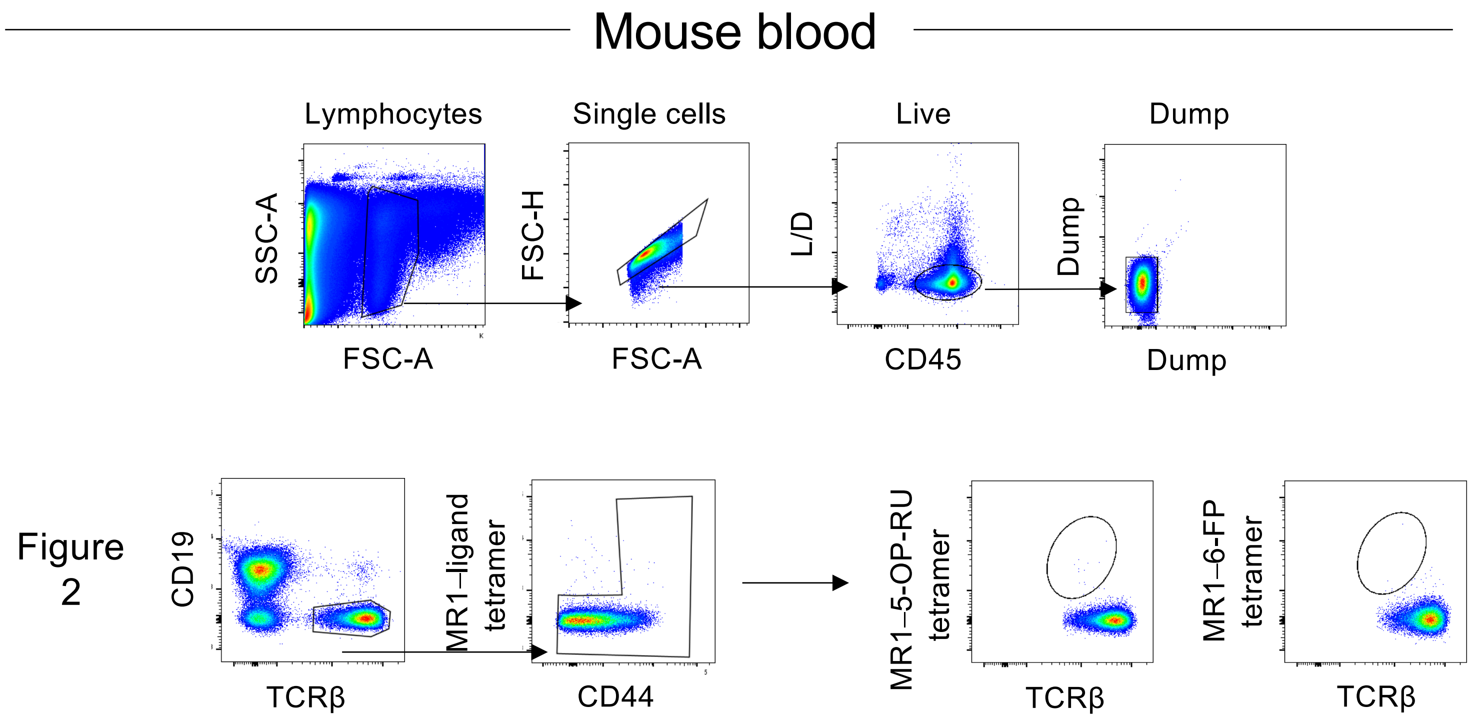

Fig. S4 Representative gating strategy for MR1–5-OP-RU and MR1–6-FP tetramer-based MAIT cell identification in mouse lungs, spleen and blood. Gating and staining strategy as depicted in Fig. 2 (species-matched MR1–5-OP-RU and MR1–6-FP tetramer MAIT cell identification) and Fig. 3 (species-mismatched MR1–5-OP-RU and MR1–6-FP tetramer MAIT cell identification) are shown.

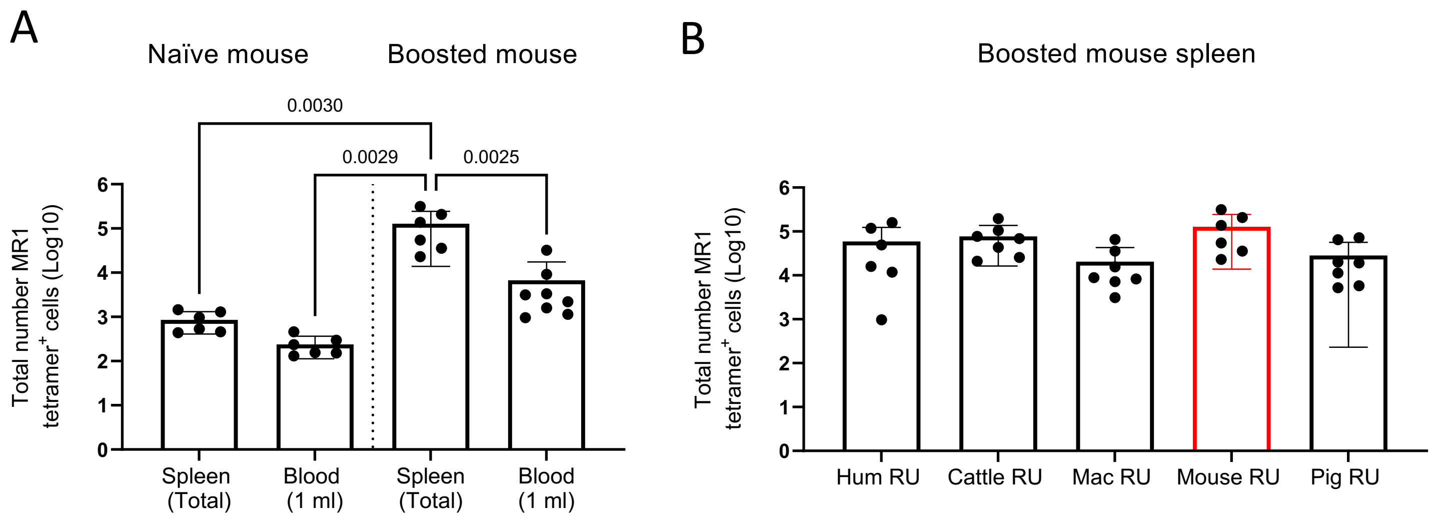

Fig. S5 Total numbers of MAIT cells in spleen and blood in naïve and in MAIT cell boosted mice. A) Total number of MAIT cells in naïve (n=6 for blood and spleen) or MAIT cell boosted mouse spleen (n=6) and whole blood (n=8). Histograms depict mean frequency ± SEM with each individual datapoint shown. Differences in total numbers obtained were evaluated using a one-way ANOVA followed by Tukey's multiple comparisons test with single pooled variance comparing staining between tissues and boosted/ unboosted mice. Only statistically significant values (p value < 0.05) are shown. Data are combined from three separate experiments. Data from the same experiments as those displayed in Fig. 2, Fig. 3 and Fig. 4.**B)** Total number of species-matched (red histograms) and species-mismatched (black histograms) MR1–5-OP-RU (RU, black dots) tetramer$^+$ cells in spleen from MAIT cell boosted mice (n=6-7). Histograms depict mean frequency ± SEM with each individual datapoint shown. Differences in total numbers obtained were evaluated using a one-way ANOVA with repeated measures, followed by Holm-Sidak's multiple comparison test, comparing staining with species-matched MR1–5-OP-RU tetramer and all other species-mismatched MR1–5-OP-RU tetramers, with individual variance computed for each comparison. No statistically significant differences (p value < 0.05) were observed. Data are combined from three separate experiments. Data from the same experiments as those displayed in Fig. 2, Fig. 3 and Fig. 4.

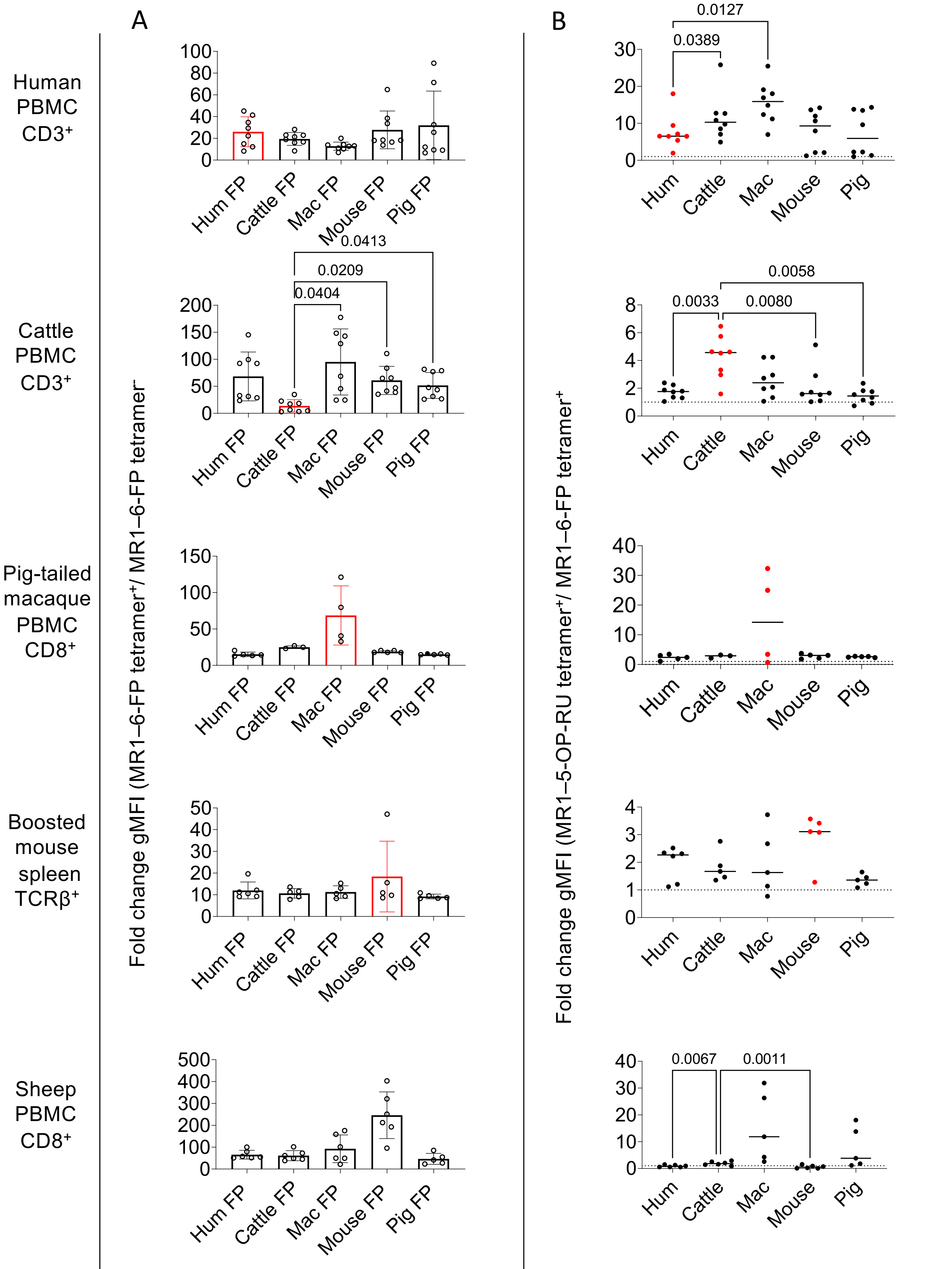

Fold change gMFI:
MR1–6-FP tetramer+/MR1–6-FP tetramer-

Tetramer / Sample	Hum	Cattle	Mac	Mouse	Pig
Hum					
Cattle					
Mac					
Mouse					

Fold change gMFI:
MR1–5-OP-RU tetramer/MR1–6-FP tetramer

Tetramer / Sample	Hum	Cattle	Mac	Mouse	Pig
Hum					
Cattle					
Mac					
Mouse					

Legend
- significant increase relative to species matched staining
- trending increase relative to species matched staining
- no change
- trending decrease relative to species matched staining
- significant decrease relative to species matched staining

Fig. S6 Fold change gMFI of the MR1–6-FP tetramer$^+$ relative to the MR1–6-FP tetramer$^-$ staining and fold change gMFI of the MR1–5-OP-RU tetramer$^+$ to MR1–6-FP tetramer$^+$ staining. Bar charts and heatmap summaries, displaying: **A**) Species-matched (red histograms) and species-mismatched (black histograms) MR1–6-FP tetramer$^+$ (FP) geometric mean fluorescence intensity (gMFI), fold change of the MR1–6-FP tetramer$^-$ population (background). **B**) Species-matched (red histograms) and species-mismatched (black histograms) fold change gMFI MR1–5-OP-RU tetramer$^+$ of gMFI MR1–6-FP tetramer$^+$ staining. Data are combined from either one (pig-tailed macaque) two (human, cattle, and sheep) or three (mouse) separate experiments. The gMFI fold change were evaluated using a one-way ANOVA with repeated measures, followed by Dunnett's multiple comparison test comparing the fold change difference between 5-OP-RU and 6-FP between species-matched and -mismatched reagents. Data are combined from three separate experiments. Data from the same experiment as that displayed in Fig. 2.

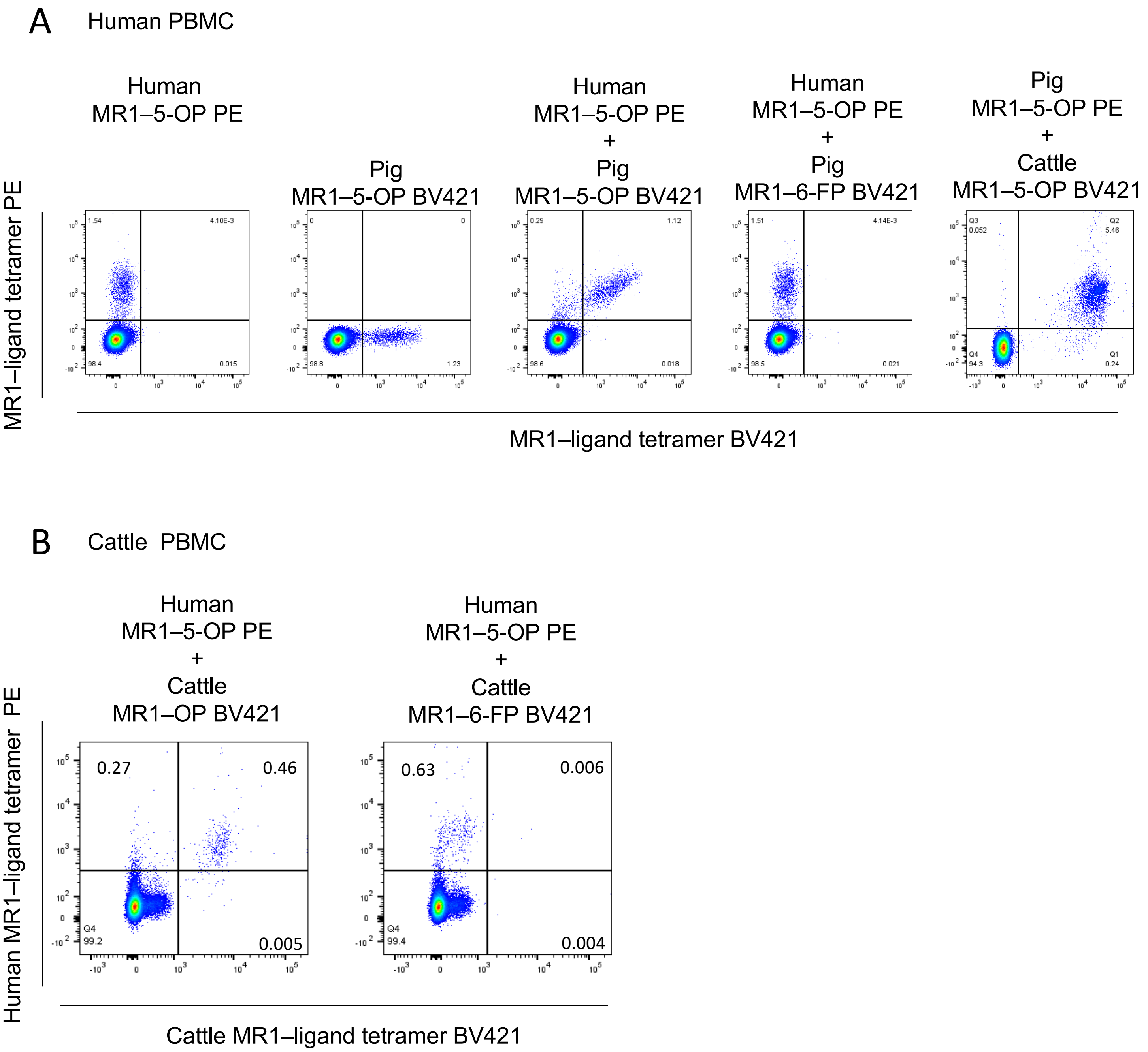

Fig. S7 Co-staining of human or cattle PBMC with species-matched and -mismatched MR1–5-OP-RU and MR1–6-FP tetramers. A) Co-staining of human PBMC with human and pig MR1–5-OP-RU (MR1–5-OP) tetramers (third panel). Human and pig MR1–5-OP-RU tetramer individual staining (1st and 2nd panels) and co-staining with human MR1–5-OP-RU and pig MR1–6-FP tetramer (4th panel) are included as controls. Human PBMC co-stained with pig and cattle MR1–5-OP-RU (MR1–5-OP) tetramers is also depicted (fifth panel). **B)** Co-staining of cattle PBMC with human and cattle MR1–5-OP-RU tetramers (first panel) or, as control, with human MR1–5-OP-RU and cattle MR1–6-FP tetramers (second panel).

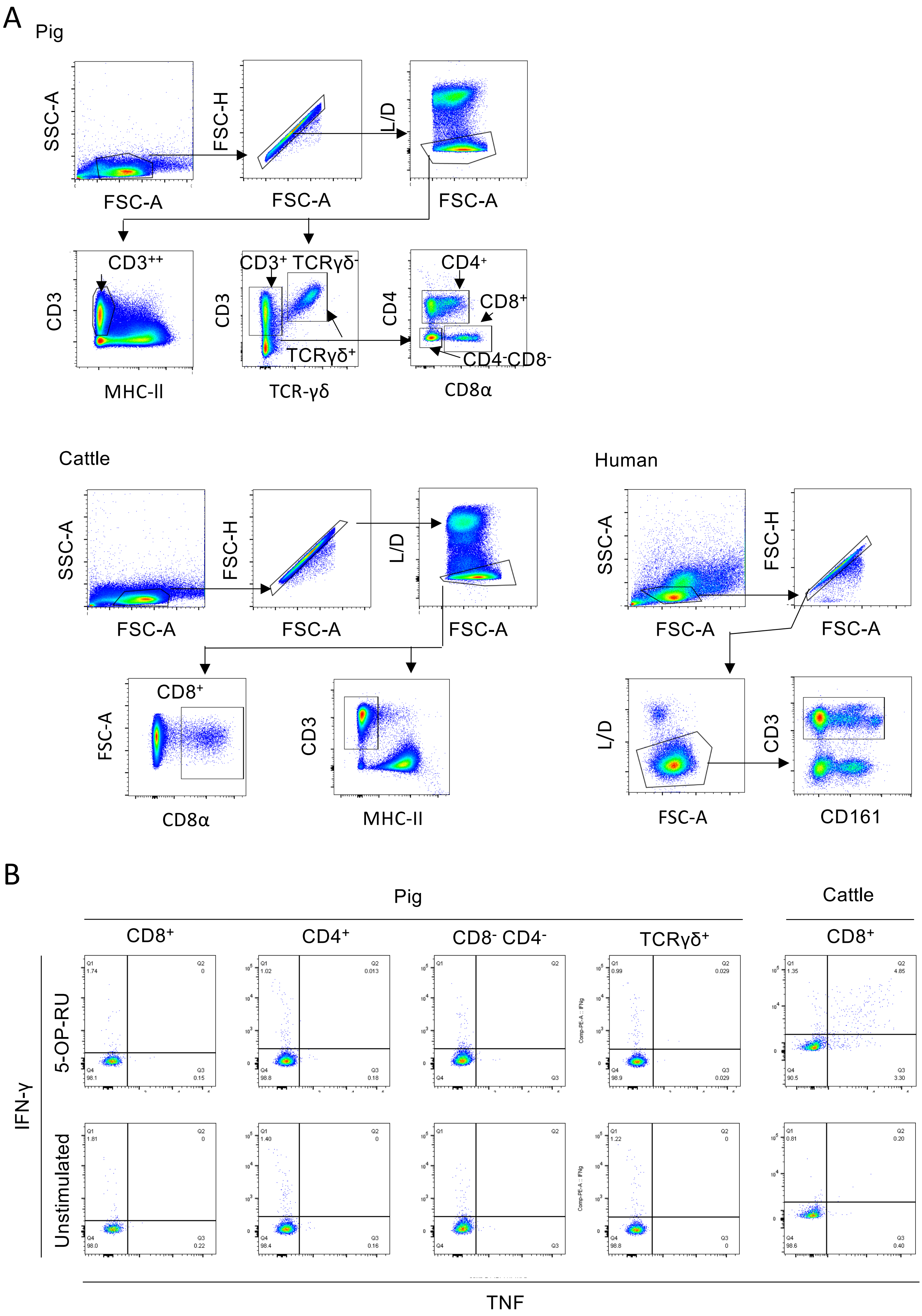
A
Pig
SSC-A
FSC-A
FSC-H
FSC-A
L/D
FSC-A
CD3
CD3++
MHC-II
CD3+ TCRγδ-
CD3
TCRγδ+
TCR-γδ
CD4+
CD4
CD8+
CD4-CD8-
CD8α
Cattle
SSC-A
FSC-A
FSC-H
FSC-A
L/D
FSC-A
CD8+
FSC-A
CD8α
CD3
MHC-II
Human
SSC-A
FSC-A
FSC-H
FSC-A
L/D
FSC-A
CD3
CD161
B
Pig
Cattle
CD8+
CD4+
CD8- CD4-
TCRγδ+
CD8+
5-OP-RU
Unstimulated
IFN-γ
TNF

C

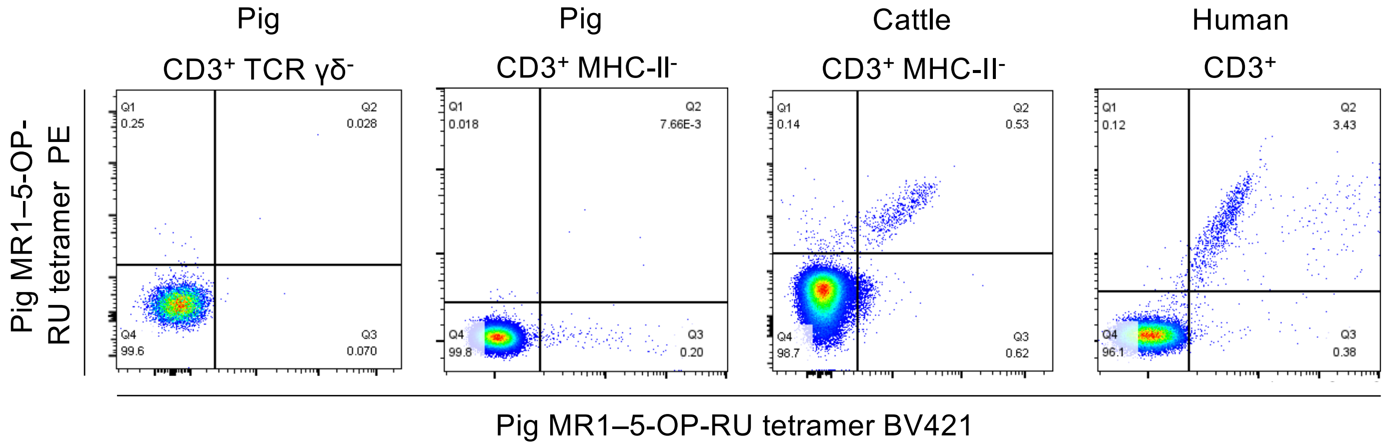

Fig. S8 Representative gating strategy for pig and cattle MAIT cell identification by MR1–5-OP-RU tetramer double staining or 5-OP-RU stimulation and intracellular cytokine staining. A) PBMC from pigs, cattle and humans were gated for distinct T cell subsets as indicated. **B)** Representative plots of intracellular cytokine staining in pig or cattle $CD8^+$, $CD4^+$, $CD8^-CD4^-$ and $\gamma\delta TCR^+$ T cells (as per the gating strategy in A), following stimulation with 5-OP-RU for 5 h or unstimulated. **C)** Representative plots for MR1–5-OP-RU tetramer double staining in pig $CD3^+$ $\gamma\delta TCR^-$ T, pig $CD3^+$ MHC-II^-, cattle $CD3^+$ MHC-II^- and human $CD3^+$ T cells (as per the gating strategy in A) with co-staining using pig-specific MR1–5-OP-RU tetramers conjugated to PE or BV421.
